# Synthesis of α-hydroxy ketones and vicinal (*R*,*R*)-diols by *Bacillus clausii* DSM 8716^T^ butanediol dehydrogenase†

**DOI:** 10.1039/d0ra02066d

**Published:** 2020-03-25

**Authors:** Lukas Muschallik, Denise Molinnus, Melanie Jablonski, Carina Ronja Kipp, Johannes Bongaerts, Martina Pohl, Torsten Wagner, Michael J. Schöning, Thorsten Selmer, Petra Siegert

**Affiliations:** Institute of Nano- and Biotechnologies, Aachen University of Applied Sciences 52428 Jülich Germany siegert@fh-aachen.de; IBG-1: Biotechnology, Forschungszentrum Jülich 52425 Jülich Germany

## Abstract

α-hydroxy ketones (HK) and 1,2-diols are important building blocks for fine chemical synthesis. Here, we describe the *R*-selective 2,3-butanediol dehydrogenase from *B. clausii* DSM 8716^T^ (BcBDH) that belongs to the metal-dependent medium chain dehydrogenases/reductases family (MDR) and catalyzes the selective asymmetric reduction of prochiral 1,2-diketones to the corresponding HK and, in some cases, the reduction of the same to the corresponding 1,2-diols. Aliphatic diketones, like 2,3-pentanedione, 2,3-hexanedione, 5-methyl-2,3-hexanedione, 3,4-hexanedione and 2,3-heptanedione are well transformed. In addition, surprisingly alkyl phenyl dicarbonyls, like 2-hydroxy-1-phenylpropan-1-one and phenylglyoxal are accepted, whereas their derivatives with two phenyl groups are not substrates. Supplementation of Mn^2+^ (1 mM) increases BcBDH's activity in biotransformations. Furthermore, the biocatalytic reduction of 5-methyl-2,3-hexanedione to mainly 5-methyl-3-hydroxy-2-hexanone with only small amounts of 5-methyl-2-hydroxy-3-hexanone within an enzyme membrane reactor is demonstrated.

## Abstract 2

Reduction of symmetric or asymmetric vicinal diketones with BcBDH leads to the synthesis of either α-hydroxyketones or vicinal diols.

## Graphical abstract

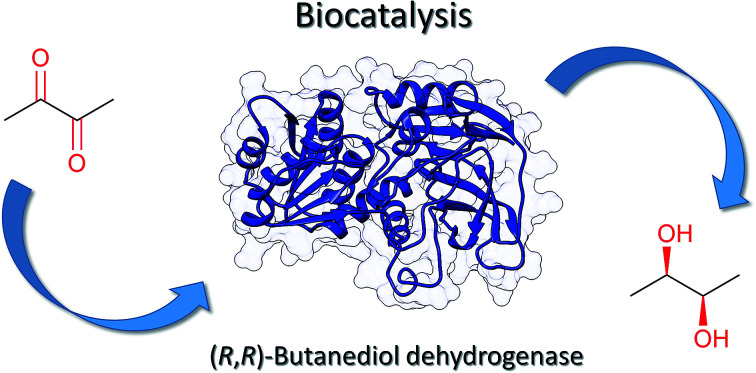

## 1 Introduction

The biocatalytic synthesis of enantiopure α-hydroxy ketones and vicinal diols is an intriguing field, due to the broad application of these molecules, *e.g.* as flavouring compounds, pheromones or as precursors for fine chemicals.^1–4^ Additionally, the development of efficient syntheses for enantiomerically enriched α-hydroxy ketones is an important research focus in the pharmaceutical industry. These compounds can be found in antidepressants and fungicides, in selective inhibitors of amyloid protein production (used in the treatment of Alzheimer's disease), in farnesyl transferase inhibitors (Kurasoin A and B), and in antitumor-antibiotics (Olivomycin A and Chromomycin A3 and Taxol).^5,6^ Several chemical approaches, like the α-hydroxylation of carbonyl compounds such as alkenes and ketone enolates, the hydrolytic kinetic resolution of terminal epoxides, or the asymmetric dihydroxylation of olefins are also reported.^7–10^ Besides the chemical synthesis, different biocatalytic routes were reported to efficiently produce α-hydroxy ketones. For example, the use of thiamine diphosphate-dependent lyases (ThDP lyases) to catalyze the carboligation of aldehydes,^11–13^ hydrolases and lipases produce α-hydroxy ketones through dynamic kinetic resolutions (DKRs)^14–16^ and redox reactions catalyzed by oxidoreductases, either by means of free enzymes (applying a cofactor regeneration system) or by whole cell biotransformations gave α-hydroxy ketones and 1,2-diols.^17–21^

Butane-2,3-diol-dehydrogenases, also known as acetoin reductases, belong to the class of oxidoreductases (EC 1.1.1.4) and catalyze the enantioselective reduction of diketones with NAD(P)H to the corresponding vicinal diols *via* an α-hydroxy ketone intermediate.^22^ Even though they are widely known to play a key role in the biological production of 2,3-butanediols,^2,23^ their biocatalytic potential is poorly studied. Nevertheless, the enantioselective synthesis of vicinal diols and α-hydroxy ketones by the butane-2,3-dehydrogenases from *Saccharomyces cerevisiae* and *Serratia marcescens* CECT 977 (ref. 18 and 19) shows the applicability of this enzyme class as biocatalyst.

Here, we report the stereoselective characteristics of our previously described enzyme, the (*R*,*R*)-butane-2,3-diol dehydrogenase from *Bacillus clausii* DSM 8716^T^,^24^ for various diketones and α-hydroxy ketones besides its physiological substrates acetoin and diacetyl.

## 2 Materials and methods

### 2.1 Chemicals and reagents

Unless stated otherwise, chemicals were analytical grade and purchased from Sigma-Aldrich. (*R*)-1-Hydroxy-1-phenylpropan-2-one ((*R*)-PAC), (*S*)-1-hydroxy-1-phenylpropan-2-one ((*S*)-PAC), (*R*)-2-hydroxy-1-phenylpropan-1-one ((*R*)-HPP), (*S*)-2-hydroxy-1-phenylpropan-1-one ((*S*)-HPP) were synthesized (M. Pohl and D. Rother, Forschungszentrum Jülich; Germany) as described elsewhere.^25,26^ 2-Hydroxy-3-methoxy-1-(4-methoxyphenyl)-1-propanone, 1-(2-chlorophenyl)-1-hydroxy-3-methyl-2-butanone and 1-(2-bromophenyl)-1-hydroxy-2-butanone were synthesized in the group of K. Zeitler (University Leipzig; Germany).

Reagents for molecular biology were from Thermo Scientific. DNA oligonucleotide synthesis and DNA sequencing were performed by Eurofins Genomics (Germany). Stargate cloning vectors and Streptactin columns were from IBA GmbH (Germany).

### 2.2 Bacterial strains and plasmids

Cloning was done in *Escherichia coli* DH5α. *Escherichia coli* BL21(DE3) was used for protein production. The Stargate® pASG.5 vector (pASG-ABC0235n-5; Ap^R^) and vector pET28a-eforRED (Km^R^, this work) were used for gene expression. Plasmid pET28-eforRED was derived from pET-28a(+) (Novagen) by introducing the *eforRED* gene encoding a red chromoprotein between the start and stop codons of the pET28a multiple cloning site. Additionally, the *eforRED* gene is flanked by *Bsa*I sites allowing golden gate cloning. The *eforRed* gene serves as stuffer DNA that is entirely replaced by the target gene upon cloning. Concurrently, the color of the colony changes from red to white indicating the cloning success. The resulting pET28a derivatives are identical to those that would be obtained by conventional cloning into the *Nco*I and *Xho*I sites of vector pET-28a(+).

### 2.3 Subcloning of BcBDH with N-terminal StrepII-tag

BcBDH production *via* autoinduction required subcloning of the gene to introduce the genetic information for an N-terminal StrepII-tag into a suitable vector. Using primers (BcBDH-fw: aaaGGTCTCccatgGCTAGCGCATGGAGTCATCCTCAATTC; BCBDH-rv: aaaGGTCTCcctcaGCTCCCTTTCTCGCCGCTAAGTTTC) the gene was amplified from plasmid pASG-ABC0235n-5 as a template. The PCR product was then introduced into vector pET28-eforRED by golden gate cloning resulting in plasmid pET28-BcBDH5′. Gene sequencing verified no changes compared to the template plasmid construct.

### 2.4 Determination of protein concentration

Protein concentration throughout enzyme purification were determined using the Bradford method and bovine serum albumin (BSA) as a standard.^27^

### 2.5 Production and purification of recombinant BcBDH in *E. coli*

Heterologous expression of the *bdhA* gene, encoding the (*R*,*R*)-butane-2,3-diol-dehydrogenase from *B. clausii* DSM 8716^T^, in *E. coli* BL21(DE3) and purification to homogeneity by affinity chromatography *via* strep-tag using Strep-Tactin® macroprep columns was done as described before,^24^ this gene was expressed by autoinduction in shake flasks using the pET28a-based expression vector. Therefore, cells were grown at 30 °C at 180 rpm in autoinduction medium for 24 h.^28,29^ Harvesting and cell disruption was done as described before.^24^ To achieve a higher biomass, the production of BcBDH in *E. coli* BL21(DE3) was carried out in 2 l-scale cultivation processes by autoinduction. Cells were grown in a defined autoinduction medium^30^ at 37 °C and 500 rpm till the diauxic shift was detectable. Then, temperature was reduced to 22 °C continuing the cultivation for further 12 h. Success of purification was controlled by SDS-PAGE and carried out according to Laemmli^31^ using 12% polyacrylamide gels and Roti-Mark PRESTAINED ladder as standard.^24^

### 2.6 Butanediol dehydrogenase activity standard assay

Enzyme reactions were followed by substrate-dependent oxidation of NADH at 340 nm over a period of 90 s using a temperature-controlled photometer (Bioscience Ultrospec 2100 Pro, Amersham). All reactions were performed at 30 °C.^24^

The reduction reactions were determined in MES–NaOH (2-(*N*-morpholino)ethane sulfonic acid) buffer (50 mM, pH 6.8) with acetoin (10 mM) and NADH (0.3 mM) as substrates. The reaction was initiated by addition of an appropriately diluted sample of purified BcBDH. A correction was made by measuring a control without enzyme. Variability is expressed as standard deviation.

One unit of BcBDH is defined as the amount of enzyme that oxidizes one μmol of NADH per minute at 30 °C under the given conditions, respectively.

### 2.7 Substrate spectrum

Unless stated otherwise, the activities toward various potential substrates were tested using standard reduction assays (Section 2.6) with 10 mM of potential substrates (individual compounds are given in the Result section). For hydrophobic compounds a final concentration of 5% dimethyl sulfoxide (DMSO) was used as a solubilizer.

### 2.8 Effect of organic solvents on the activity of BcBDH

The stability of the purified enzyme toward organic co-solvents was tested with or without the addition of organic co-solvents (v/v), respectively (individual compounds are given in the Result section) in Tris–HCl buffer (10 mM; pH 7.4) supplemented with NaCl (150 mM). Residual activities were measured after 1 h incubation at 22 °C using the acetoin reduction assay (see Section 2.6).

### 2.9 Effect of metal ions and EDTA on the activity of BcBDH

The effect of different metal ions on BcBDH activity was determined by adding a final concentration of 1 mM of different metal salts (CoCl_2_·6H_2_O, FeCl_2_, NiSO_4_·6H_2_O, MgCl_2_, MnCl_2_, KCl and ZnSO_4_) to the enzyme preparation without any further pretreatment. The activity without supplement was defined as 100%. In order to remove divalent metal ions from BcBDH 1 mM EDTA (ethylenediaminetetraacetic acid) was added. After incubation for 1 h at 22 °C the residual activity was determined using the standard reduction assay as described above.

To elucidate the effect of metal ion supplementation over a longer period of time, BcBDH was incubated for 25 hours at 22 °C in its storage buffer Tris–HCl (10 mM; pH 7.4) with NaCl (150 mM), supplemented with MnCl_2_ and ZnSO_4_ (1 mM each), respectively. The reference was prepared in the same way without metal ion addition. Activity was measured after 1, 2, 4, and 25 hours with the standard photometric assay. The activity without supplement was defined as 100%.

### 2.10 Stereoselectivity of BcBDH by GC

Stereoselectivity and conversion was analyzed by carrying out the reduction reaction of selected diketones and α-hydroxy ketones (see Section 3.5). Formate dehydrogenase (FDH) from *Candida boidinii* (Megazyme) was used for cofactor regeneration. Biotransformations were carried out in 1.5 ml Eppendorf vials at 30 °C for 60 min without agitation in a total volume of 1 ml. The standard reaction mixtures for the reduction reaction consisted of: substrate (10 mM), purified BcBDH (1 U ml^−1^ of the corresponding substrate), FDH (5 U ml^−1^), sodium formate (30 mM) and NADH (0.3 mM) in MES–NaOH buffer (50 mM; pH 6.8). Samples (100 μl) were taken at different points in time during the reaction, extracted with diethyl ether (300 μl) and applied to GC analysis (GC-2010 Plus (Shimadzu) with a flame ionization detector) equipped with a Hydrodex γ-DIMON (25 m × 0.25 mm ID Macherey & Nagel) column. The following temperature profile was used: 45 °C (2 min), 45–70 °C (at 2 °C min^−1^); 70–180 °C (at 10 °C min^−1^); 180 °C (10 min). For benzoin analysis the last step was extended to 180 °C (60 min).

Retention times of educts (as standards purchased from Sigma-Aldrich or synthesized) were: diacetyl (2,3-butanedione) 4.1 min; (*R*)-acetoin 11.7 min; (*S*)-acetoin 14.4 min; racemic 4-hydroxy-3-hexanone 18.6 and 19.7 min; 2,3-pentanedione 9.8 min; 3-hydroxy-3-methyl-2-butanone 10.5 min; 2,3-hexanedione 12.9 min; 3,4-hexanedione 15.9 min; 5-methyl-2,3-hexanedione 13.3 min; methylglyoxal (2-oxopropanal) 2.3 min; (*S*)-2-hydroxy-1-phenylpropan-1-one (HPP) 24.9 min; (*R*)-2-hydroxy-1-phenylpropan-1-one 24.7 min; (*S*)-1-hydroxy-1-phenylpropan-2-one (PAC) 24.8 min; (*R*)-1-hydroxy-1-phenylpropan-2-one 25.0 min, benzoin 43.0 min.

Retention times of the products were (as standards purchased from Sigma-Aldrich or BcBDH synthesized and identified as shown in Section 3.5): (*S*,*S*)-butane-2,3-diol 17.6 min; (*R*,*R*)-butane-2,3-diol 17.8 min; *meso*-butane-2,3-diol 18.1 min; 3-hydroxy-2-pentanone 16.4 min; 2,3-pentanediol 19.8 min; (*R*)-3-hydroxy-2-hexanone 19.3 min; (*R*)-2-hydroxy-3-hexanone 19.4 min; (*R*)-3,4-hexanediol 21.0 min; (*R*)-5-methyl-2-hydroxy-3-hexanone 19.7 min; (*R*)-5-methyl-3-hydroxy-2-hexanone 19.9 min; 2-methyl-2,3-butanediol 17.8 min; 3,4-hexanediol 21.0 min; (*R*,*S*)-1-phenyl-1,2-propanediol 27.2 min; (*R*,*R*)-phenyl-1,2-propanediol 27.0 min; (*S*,*S*)-phenyl-1,2-propanediol 26.9 min.

### 2.11 Chemical synthesis of diols

Standards of 2,3-pentanediol, 2,3-hexanediol, 3,4-hexanediol, and 5-methyl-2,3-hexanediol were obtained by reduction of the corresponding diketones (2 mmol) with sodium borhydride (NaBH_4_, 2 mmol) in 20 ml methanol. The reaction mixture was incubated for 3 hours 20 °C under stirring. After the slowly addition of 1 ml 10% HCl and 30 ml H_2_O the reaction was stirred for another 10 min. The reaction products were extracted with diethyl ether, neutralized, dried with MgSO_4_ (anhydrous), filtrated and the solvent was removed in vacuum.^18^

### 2.12 Preparative biocatalytic synthesis

Preparative biotransformations of 2,3-pentanedione, 2,3-hexanedione, 3,4-hexanedione and 5-methyl-2,3-hexanedione were carried out in a volume of 20 ml. The reaction mixture consisted of diketone (200 mM), BcBDH (0.5 U ml^−1^), formate dehydrogenase (FDH) for cofactor regeneration (0.8 U ml^−1^), sodium formate (600 mM), NADH (0.3 mM) and 10% methanol in MES–NaOH buffer (50 mM; pH 6.8). The reaction was incubated at 30 °C for 48 hours under gentle stirring. The products were extracted with diethyl ether, dried with MgSO_4_ (anhydrous), filtrated and the solvent removed by distillation and analysed by GC-MS.

The BcBDH reduction product of 5-methyl-2,3-hexanedione was additionally analysed by ^1^H and ^13^C nuclear magnetic resonance (NMR; NMR spectrometer Spinsolve 60 carbon (60 MHz) (Magritek)). The sample was solved in 1 ml CDCl_3_. ^1^H-NMR (CDCl_3_) *σ* 0.90 (d, 3H), 1.00 (d, 4H), 1.43 (ddd, 2H), 1.95 (m, 1H), 2.17 (s, 4H), 3.20 (s, 2H 4.18 (m, 1H).

### 2.13 GC-MS analysis

Samples were analyzed by GC-MS (GC-2010 Plus (Shimadzu) with flame ionization detector coupled with a quadrupole-mass spectrometer (GC-MS-QP2010S, Shimadzu). Molecule fragmentation was achieved by electron ionization (70 eV).

### 2.14 Circular dichroism

Circular dichroism (CD) spectroscopy of different products was performed using a Jasco J-1100 spectrometer with a 1 mm optical path length cell. The extracted biocatalytic products were appropriately diluted in ethanol. The ellipticity readout were converted to molar ellipticity units with the following eqn (1):^32^1
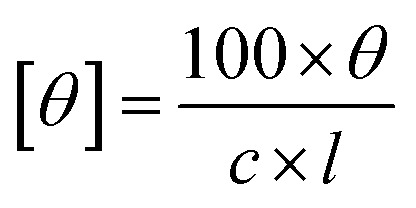
Here, [*θ*] = molar ellipticity in deg × cm^2^ × dmol ^−1^; *c* = concentration of an optical active compound in M; *l* = cuvette optical path length in cm.

### 2.15 Synthesis of (*R*)-5-methyl-3-hydroxy-2-hexanone with an enzyme membrane reactor

Biotransformation with the enzyme membrane reactor were carried out using a stainless steel reactor with a volume of 10 ml with an internal magnetic stirrer, coupled with a thermostat for temperature control, a bubble trap, a sterile filter, a 10 kDa ultrafiltration membrane (Ultracel® Merck; regenerated cellulose), a HPLC pump and a sampler. The reaction mixture consisted of: 5-methyl-2,3-hexanedione (100 mM), BcBDH (20 U), FDH (60 U), formate (300 mM), NADH (0.3 mM) and 5% methanol in MES–NaOH buffer (50 mM; pH 6.8) in a volume of 300 ml. The flow rate was set to 0.2 ml min^−1^. The reactor outflow was fractionated (10 ml samples) and analysed by GC.

## 3 Results and discussion

### 3.1 Cloning and expression of the (*R*,*R*)-butane-2,3-diol-dehydrogenase

The cloning of the (*R*,*R*)-butane-2,3-diol dehydrogenase gene from *B. clausii* DSM 8716^T^ (BcBDH) in a pASG vector was described in our previous publication.^24^ In addition, the BcBDH gene with N-terminal StrepII-tag was cloned into a pET28a expression vector allowing expression by autoinduction. By using this system, no differences in BcBDH expression in shake flasks and 2 l fermentations were observed, but a higher biomass production and therefore more enzyme was achieved. After affinity chromatography purification, the enzyme was essentially pure with a specific activity of 70–100 U mg^−1^.^24^

### 3.2 Extended substrate specificity of BcBDH

As reported in our previous publication BcBDH has the ability to reduce α-hydroxy ketones, vicinal diketones, and hydroxyaldehydes, only^24^ (Table 1; 1–9). Besides its natural substrates diacetyl and acetoin, BcBDH accepts several non-physiological molecules as substrates and is also able to catalyze the oxidation reaction, but solely of vicinal diols to the corresponding α-hydroxy ketones. No oxidation of primary or secondary alcohols could be detected.

**Table 1:** Substrates used for activity studies. Enzyme activities were measured with the standard reduction assay and compared to acetoin. 100% = 70–100 U mg^−1^

Substrate	Activity [%] (photometric assay)	Substrate	Activity [%] (photometric assay)
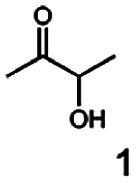	100	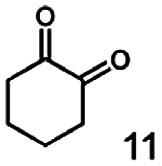	0
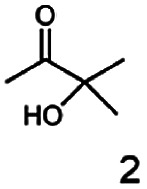	86.4 ± 3.1	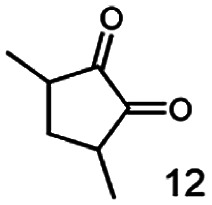	0
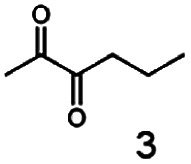	59.2 ± 3.4	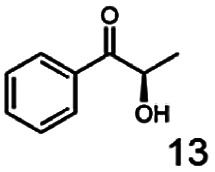	194.6 ± 8.7
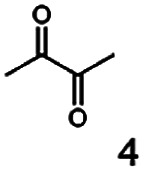	55.7 ± 0.9	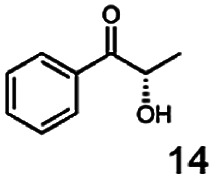	111.5 ± 4.9
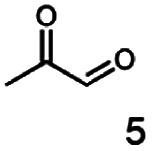	54.0 ± 5	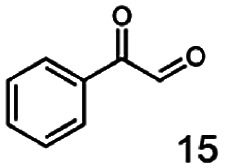	46.8 ± 3.5
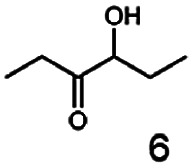	48.5 ± 3.3	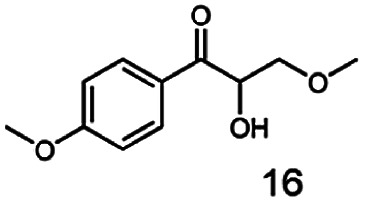	7.1 ± 0.4
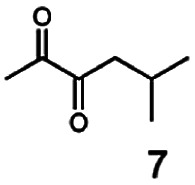	44.3 ± 2.3	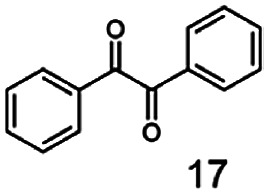	0
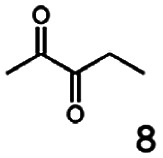	38.9 ± 2.1	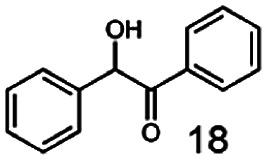	0
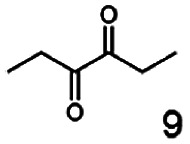	15.6 ± 0.6	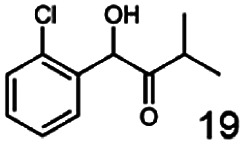	0
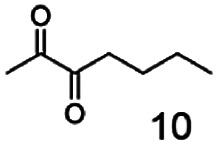	93 ± 9	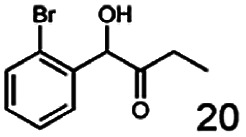	0
		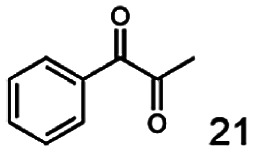	52.4 ± 5.3
		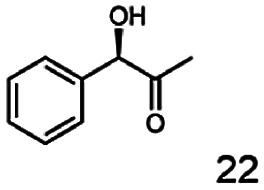	0
		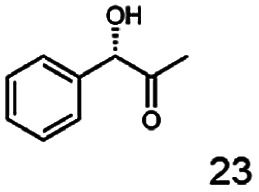	0

In this study we further elucidated the substrate scope of the enzyme. Additional molecules with a branched aliphatic structure or with a phenyl moiety were probed (7, 10–23). Activity tests were carried out as described before (10 mM substrate; 50 mM MES–NaOH; 0.3 mM NADH; pH 6.8 at 30 °C). Surprisingly the enzyme also exhibited some remarkable activity for some substrates which contain a phenyl moiety or a bulky aliphatic residue: 2,3-heptanedione (10), 5-methyl-2,3-hexanedione (7), (*R*)-HPP (13), (*S*)-HPP (14) 1-phenyl-1,2-propanedione (21), phenylglyoxal (15), 2-hydroxy-3-methoxy-1-(4-methoxyphenyl)-1-propanone (16), 1,2-cyclohexanedione (10). The compounds 3,5-dimethyl-1.2-cyclopentanedione (11), benzil (17), benzoin (18), 1-(2-chlorophenyl)-1-hydroxy-3-methyl-2-butanone (19), 1-(2-bromophenyl)-1-hydroxy-2-butanone (20) are no substrates of BcBDH. Surprisingly under these conditions the activity of BcBDH toward (*R*)-2-hydroxy-1-phenylpropan-1-on ((*R*)-HPP), a substrate with a phenyl moiety, is much higher than toward its natural substrate racemic acetoin. (*S*)-2-hydroxy-1-phenylpropan-1-one ((*S*)-HPP) is converted with 111.5% activity compared to acetoin. On the contrary no activity for both enantiomers of 1-hydroxy-1-phenylpropan-2-one (PAC; 22, 23) was observed (Table 1). This is the first time a (*R*)-butane-2,3-diol dehydrogenase is described to accept these aromatic molecules as substrates. In contrast to that, substrates with two phenyl moieties like benzil and benzoin, are not converted. Of particular interest is that substrates with at least one keto group next to the phenyl moiety, like HPP (13/14) or 2-hydroxy-3-methoxy-1-(4-methoxyphenyl)-1-propanone (16), are accepted, but not substrates with an external keto group (*e.g.* PAC (22), 1-(2-chlorophenyl)-1-hydroxy-3-methyl-2-butanone (19), 1-(2-bromophenyl)-1-hydroxy-2-butanone) (20). The activity of BcBDH with both HPP enantiomers but not with the PAC enantiomers underlines the suggestion that this enzyme prefers α-hydroxy ketones with the ketone group adjacent to a phenyl ring. While the reduction of “bulky” diketones or α-hydroxy ketones is new for (*R*)-butane-2,3-diol dehydrogenases, there are already several oxidoreductases reported for the reduction of “bulky” aliphatic and aromatic α-hydroxy ketones, diketones and ketones.^33–36^

### 3.3 Influence of metal salts and EDTA on BcBDH activity

**Fig. 1:**
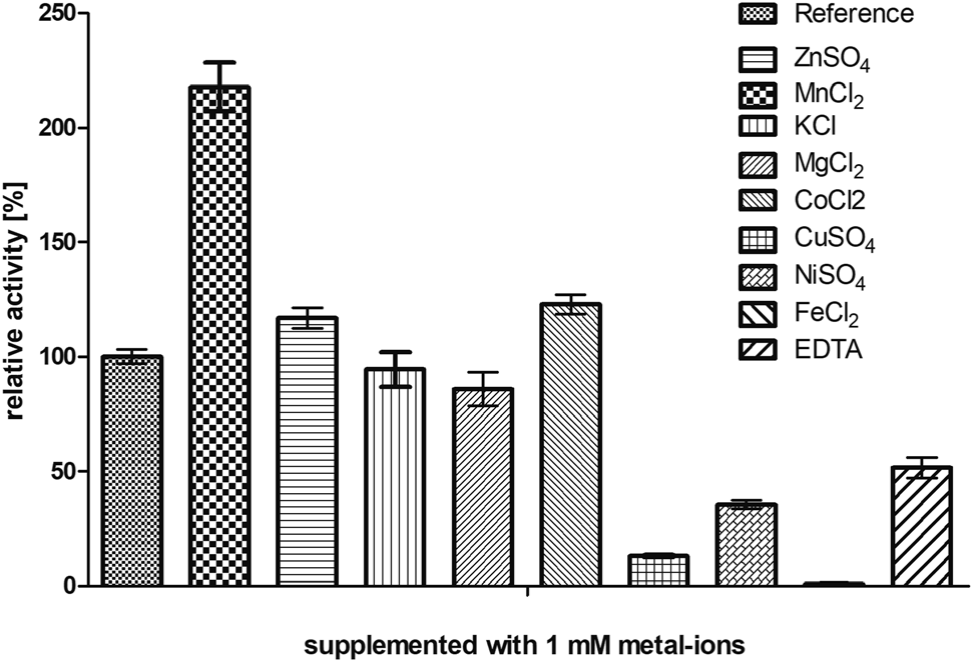
Influence of several metal ions and the chelator EDTA on the enzyme activity. BcBDH was incubated in Tris–HCl buffer (10 mM; pH 7.4) with NaCl (150 mM) supplemented with several metal salts and EDTA (1 mM) for 1 hour at 22 °C. Activity was measured with the standard photometric assay. Reference was treated the same way without any addition.

The enzyme BcBDH belongs to the superfamily of medium-chain dehydrogenases/reductases (MDR). Butane-2,3-diol dehydrogenases/acetoin reductases of that superfamily are mostly Zn^2+^-dependent and need at least one catalytical zinc ion, bound to the highly conserved catalytic amino acids Cys_37_-His_70_-Glu_71_ (BcBDH numbering). Additionally, some enzymes of that superfamily bind a structurally relevant zinc ion.^37,38^ For that reason, we investigated the influence of Zn^2+^ and other bivalent metal ions on BcBDH activity. Purified BcBDH without further pretreatment EDTA (note that the purification protocol is without addition of ZnCl_2_) incubated with 1 mM of the respective metal salts (22 °C, Tris–HCl buffer, 10 mM; pH 7.4) and the residual activity (standard reduction assay) was determined after one hour. As shown in Fig. 1 the metal ions strongly influenced the enzyme's activity. A loss of activity was detected with NiSO_4_ (65%), with CuSO_4_ (87%) and with FeCl_2_ (100%). In contrast to that, KCl and MgCl_2_ showed no effect. Most interestingly, addition of ZnSO_4_ only slightly increased BcBDH activity (116%), whereas the addition of cobalt ions (CoCl_2_: 122%) and manganese-(ii)-ions (MnCl_2_: 217%) strongly activated BcBDH. As expected, the incubation of this enzyme with the chelator EDTA led to an activity loss (50%). By using EDTA for chelating the Zn^2+^ ions a complete inactivation of this enzyme was not possible under the applied conditions. Additionally, no activity loss of this enzyme during purification without Zn^2+^ ions was determined.

**Fig. 2:**
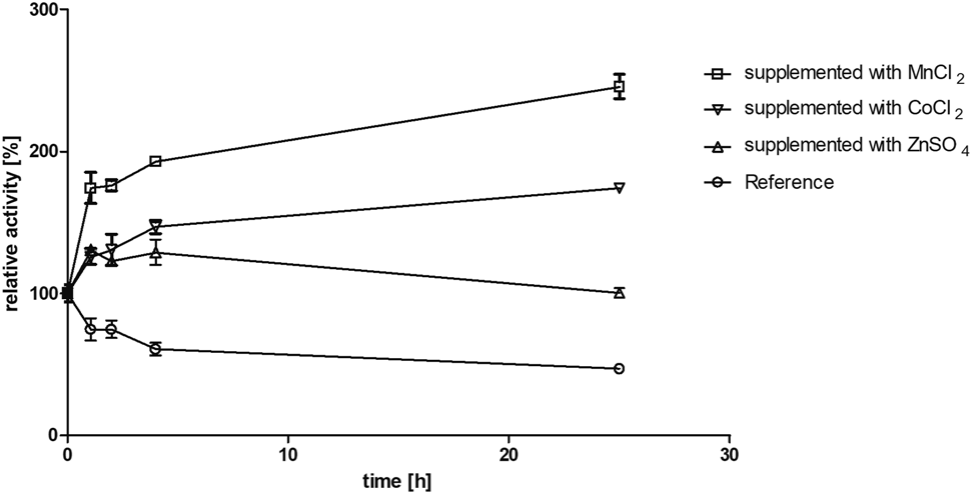
Effect of MnCl_2_, CoCl_2_ and ZnSO_4_ (1 mM each) on incubation in Tris–HCl buffer (10 mM; pH 7.4, 150 mM NaCl) for 25 hours at 22 °C. Activity was measured after 1, 2, 4, 25 hours with the standard photometric assay. Reference was treated the same without metal ion addition.

Motivated by the observed activation we further investigated the effect of manganese(ii) and cobalt(ii) ions on BcBDH for biocatalytical purposes. To elucidate the kinetics of that effect BcBDH (without any further pretreatment) was supplemented with 1 mM MnCl_2_ and CoCl_2_, respectively, and the activity was compared to a reference sample for 25 hours (22 °C). BcBDH without any supplement (reference sample) rapidly lost activity over time. After 25 hours the residual activity was only 46% of the initial value. In contrast to that, BcBDH supplemented with 1 mM CoCl_2_ or MnCl_2_ showed an increase in activity over time. The supplementation with Co^2+^ ions led to an activity increase of 174%, but the addition of Mn^2+^ ions more than doubled the activity (245%) after 25 hours. Supplementation with Zn^2+^ ions showed only a slight activity increase after the first hour (130%) and resulted into an enhanced stability of this enzyme but no further activation with an activity of 100% after 25 hours (Fig. 2). For oxidation of (*R*,*R*)-2,3-butanediol and *meso*-butanediol (10 mM each; 0.3 mM NAD^+^; 50 mM Tris–HCl pH 8.0; 30 °C) the same effect was observed which resulted in a 3-fold higher enzyme activity and an enhanced stability (data not shown). (*S*)-Acetoin is still not a substrate of Mn^2+^ “substituted” BcBDH.

Moreover, we investigated the influence of Zn^2+^ and Mn^2+^ ion addition on biocatalysis. The enzyme was preincubated with Zn^2+^ and Mn^2+^ ions, respectively, as described in Section 2.9. The reference was incubated with addition of the same amount of buffer without metal salts to maintain the same enzyme concentration. The same volumes of untreated (reference) BcBDH and of metal ion preincubated enzyme were used. The activity enhancement of Mn^2+^-supplemented BcBDH could also be observed in biotransformation experiments leading to a higher conversion rate. Whereas a total conversion of racemic acetoin (10 mM, pH 6.8, 30 °C, untreated BcBDH 0.5 U ml^−1^) to (*R*,*R*)-BD without metal ion addition could be achieved within 40 minutes, conversion BcBDH supplemented with 1 mM Mn^2+^ was already completed after 10 minutes. The supplementation of Zn^2+^ ions led to no effect in comparison to the reference. The increase of activity, by Mn^2+^ and other bivalent transition metal ions, was already reported for other dehydrogenases (*e.g.* glycerol dehydrogenase, alcohol dehydrogenase from *Thermoanaerobacter brockii*) but is new for BDHs.^39–41^ Another study showed that the substitution of the zinc-dependent *Thermoanaerobacter brockii* ADH with Co^2+^ leads to an oxidation of the ion while forming an octahedral (instead of a tetrahedral like Zn^2+^) structure complex at the active site. The higher valence of the metal ion may contribute to enhanced substrate binding and reaction intermediate stabilization.^42^ Furthermore, it could be shown that the substitution of the catalytic zinc ion in yeast ADH by cobalt or copper led to an enhancement of some biochemical properties, like thermo stability and pH stability.^43,44^ The results shown here indicate that metal ion substitutions could be applied to improve the catalytic properties of BcBDH. Amino acid sequence analysis revealed no additional binding motives for Mn^2+^ ions which could probably explain the activation. We suggest that the catalytic zinc ion is substituted over time by the Mn^2+^ ion. However, this is an intriguing effect and more research is needed to gain a deeper mechanistic insight.

Mutagenesis experiments involving the catalytic amino acids C37, H70 and E151 (C37A, H, S; H70A, C, Q; E151A, C, Q), which are crucial for binding the catalytic zinc ion, resulted in the loss of expression or in inactive enzyme variants (data not shown). Same results were published for the alcohol dehydrogenase of *Thermoanaerobacter brockii*.^41^ Here, the mutagenesis of the catalytic amino acids led to inactive variants, too.

### 3.4 Effect of organic solvents on BcBDH activity

**Fig. 3:**
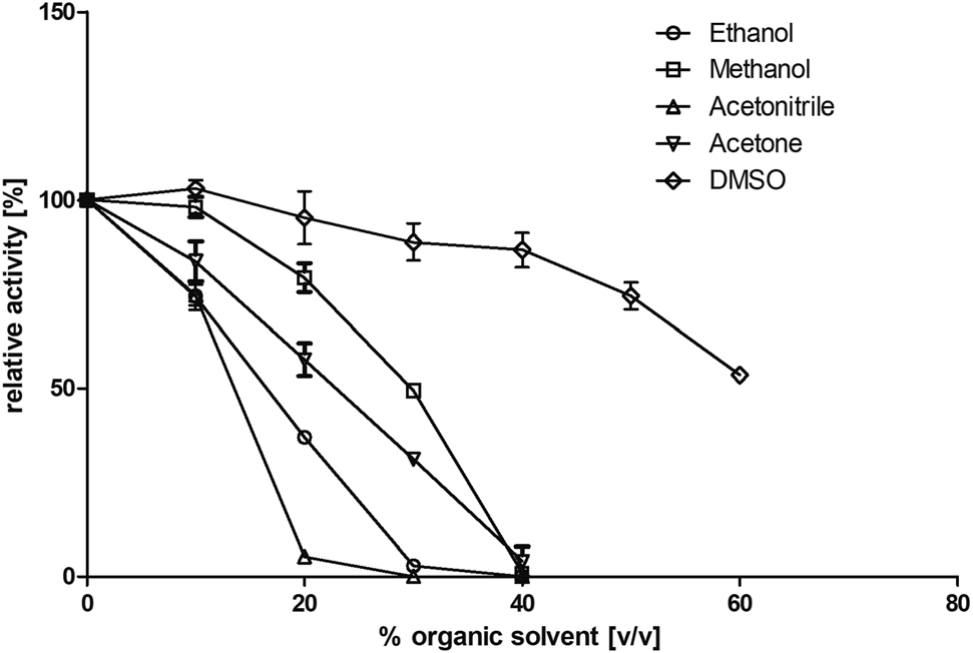
Influence of several water miscible organic solvents on enzyme activity. BcBDH was incubated for 1 hour (22 °C) with 0 to 60% (v/v) solvent in Tris–HCl buffer (10 mM; pH 7.4) with NaCl (150 mM). Activity was measured with the standard photometric assay.

Residual activity (standard assay) was tested after addition of 0 to 60% (v/v) of different solvents after 1 hour of incubation (22 °C; Tris–HCl buffer, 10 mM; pH 7.4 with NaCl, 150 mM). The following organic solvents were tested: dimethyl sulfoxide (DMSO), acetone, methanol, ethanol, and acetonitrile (Fig. 3). It turned out that this enzyme shows its highest tolerance toward DMSO. Addition of up to 60% (v/v) DMSO resulted in a residual activity of 53% after incubation. In contrast, BcBDH seems to be quite sensitive toward acetonitrile and ethanol. A nearly complete loss of activity after the addition of 30% (v/v) of these organic solvents was determined. Organic solvent resistant enzymes are preferred for practical biocatalytic purposes. Until now, butanediol dehydrogenases are not well studied in that respect. The (*R*,*R*)-2,3-butanediol dehydrogenase from *Rhodococcus erythropolis* WZ010, for example, showed a good resistance against DMSO with a relative activity of 84% by adding 30% (v/v) DMSO to the assay (but without pre-incubation), but loses much activity at higher concentrations.^45^ On the other hand, there are alcohol dehydrogenases, *e.g.* from *Paracoccus pantotrophus* DSM 11072 (up to 50% (v/v) DMSO) and *Rhodococcus ruber* DSM 44541 (*e.g.* up to 50% (v/v) in acetone and 80% (v/v) in 2-propanol) reported, which perform extremely well in aqueous/organic solvents or even micro aqueous-systems.^46,47^

For the biocatalysis of hydrophobic substrates we decided to use methanol as a solubilizer, because of its low boiling point and therefore easier evaporation from preparative-scale biocatalytic samples, and the enzyme's good tolerance toward this solvent.

### 3.5 Biotransformations

The stereoselective synthesis of α-hydroxy ketones and vicinal diols is an intriguing field, because of the broad applications of these substances. They can be used either as natural flavours, pheromones, or as building blocks in organic syntheses.^3,4,48^ Often, it is beneficial to synthesize these molecules through biocatalysis, because of the helpful properties of enzymes (*e.g.* regio- and stereoselectivity) as biocatalysts. The butanediol dehydrogenase from *B. clausii* is (*R*)-selective toward its physiological substrate diacetyl, which leads to the vicinal diol (*R*,*R*)-2,3-butanediol *via* (*R*)-acetoin. Racemic acetoin is converted to *meso*- and (*R*,*R*)-2,3-butanediol by BcBDH. Oxidation of (*R*,*R*)-2,3-butanediol and *meso*-butanediol lead to (*R*)- and (*S*)-acetoin, respectively.^24^ Acetoin is not a substrate of BcBDH with respect to the oxidation reaction. Nevertheless, there is still the unsolved question whether this enzyme exhibits the same stereoselectivity for its unphysiological substrates, which we investigated with the following experiments. The reduction of vicinal diones and hydroxy ketones was performed in small batch experiments with 10 mM substrate.

Starting from a diketone a dehydrogenase catalyzed reduction may lead to up to four α-hydroxyketone intermediates and subsequently to up to four diols (or three α-hydroxy ketones and diols if *meso*-forms are possible). To elucidate that question we investigated the selectivity of BcBDH for various α-diketones and α-hydroxy ketones. In the case of the molecules 2, 3, 6, 7, 10, 13, 14, 15 a total conversion after one hour could be achieved. For molecules 8, 9 and 21 a conversion of 90%, 88% and 97% after one-hour reaction time was achieved. No conversion of 2-hydroxy-3-methoxy-1-(4-methoxyphenyl)-1-propanone (16; 5% methanol (v/v)) could be detected. Although, a low activity for this substrate in the photometric assay was found. Probably, the addition of methanol as solubilizer led to a further decrease of the enzyme's activity for this already poorly accepted molecule. Even though no activity was detected for benzoin (5% DMSO (v/v)) in the photometric assay, biotransformation was performed to see whether some conversion could be detected over a prolonged period of time. However, no conversion took place.

Furthermore, this enzyme revealed a high selectivity for several substrates 2, 9, 10, 13, 14, which resulted in the formation of only one reduction product. In the batch reactions of 3 and 8 traces of a second product were present and in the case of 7 also a third product was detectable in traces. Reduction of phenylglyoxal (15) resulted in the formation of three products. The reduction of racemic 6 led to the generation of two diol products, of which one has the same retention time in GC, like the diol generated by the reduction of 3,4-hexanedione. The product formed by the reduction of substrate 13 could be identified as (*R*,*R*)-phenyl-1,2-propanediol by the use of a respective GC analysis (compared to standard). The retention time of the product formed by the reduction of compound 14 could neither be matched with the retention times of (*R*,*R*)-phenyl-1,2-propanediol nor (*S*,*S*)-phenyl-1,2-propanediol. Therefore, we presume the formation of (*R*,*S*)-phenyl-1,2-propanediol.

The reduction of 21 led to the generation of mainly (*R*)-1-hydroxy-1-phenylpropan-2-one ((*R*)-PAC) with (*S*)-1-hydroxy-1-phenylpropan-2-one ((*S*)-PAC) as a side product (ee: 86%).

Besides these three molecules no suitable references of the potentially produced diols of the here tried molecules were obtainable. To overcome this limitation, we reduced the diketones with NaBH_4_ to obtain the racemic diols as a reference. To further investigate the stereoselectivity of the enzyme for aliphatic molecules, we focused on biotransformations of compounds 3, 7, 8, 9, 10, because of the good performance BcBDH demonstrated for these substrates and in order to use a variety of aliphatic (symmetric, asymmetric, branched) molecules. The retention times of the chemically synthesized diols were compared to the products of the biotransformations. Combining this with the GC-MS analysis, we could verify that asymmetric molecules like, 8, 3, 7, are surprisingly reduced to the corresponding α-hydroxy ketones (see ESI†). By reduction of 8 we obtained 3-hydroxy-2-pentanone. Reduction of molecule 3 resulted in of 3-hydroxy-2-hexanone with small amounts of 2-hydroxy-3-hexanone. Reduction of 10 gave solely the 3-hydroxy-2-heptanedione, and reduction of 7 resulted mainly in 5-methyl-3-hydroxy-2-hexanone with small amounts of 5-methyl-2-hydroxy-3-hexanone and traces of its isomer of 5-methyl-3-hydroxy-2-hexanone (Table 2 and ESI†) was detected. No further reduction of molecules 3, 7, 10 to the corresponding diols was observed (within the reaction time of one hour and the chosen conditions). Only for substrate 8, with the smallest aliphatic chain of the tested molecules, traces of the diol were detected, too. On the other hand, the reduction of the symmetric molecule 9 resulted in the generation of only one 3,4-hexanediol, but no α-hydroxy ketone intermediate was detectable over the reaction time (Table 2). It seems the enzyme is able to distinguish between symmetric and asymmetric molecules, which induces the generation either of a diol, or in the latter case, of an α-hydroxy ketone.

**Table 2:** Biocatalytic conversion of α-diketones and α-hydroxy ketones substrates by (*R*,*R*)-BDH of *B. clausii* and comparison to the specific activity toward reduction of acetoin. Substrates (10 mM) were reduced by 1 U ml^−1^ BDH using formate (30 mM) and formate dehydrogenase (5 U ml^−1^) to regenerate NADH (0.3 mM) at 30 °C in MES–NaOH buffer (50 mM, pH 6.8; 1 ml, 60 min). Data for acetoin and diacetyl data were taken from Muschallik *et al.*^24^

Substrate	Biocatalytic conversion [%]	Identified products	ee/de [%]
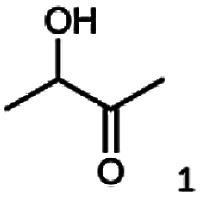	99	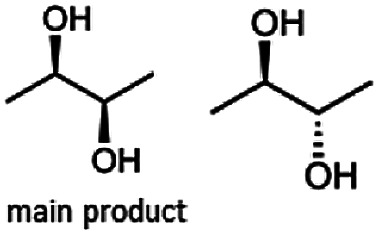	11
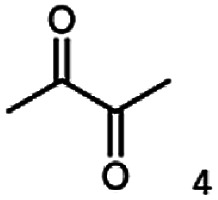	99	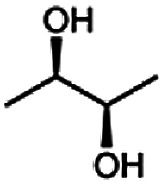	99
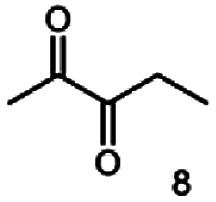	90	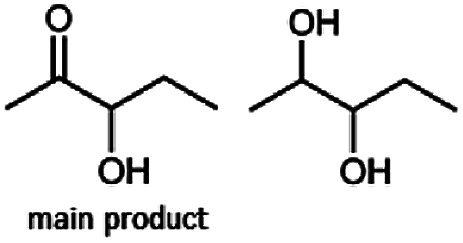	—
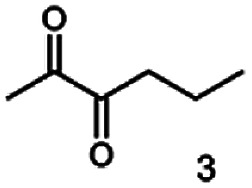	99	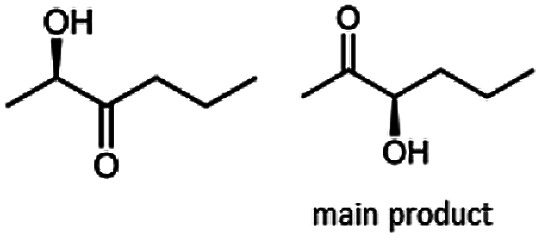	89
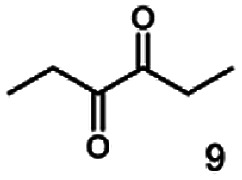	88	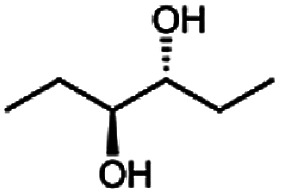	99
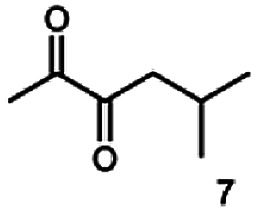	99	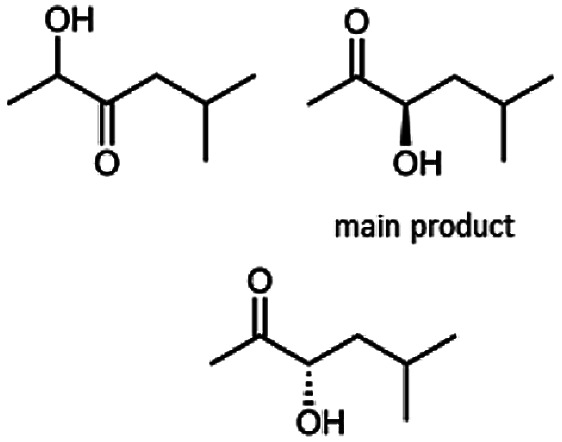	97
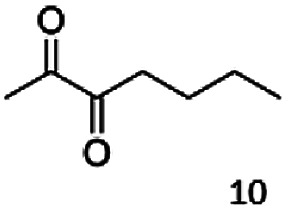	99	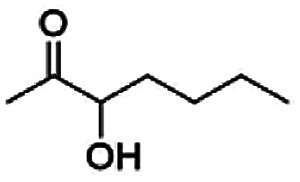	99
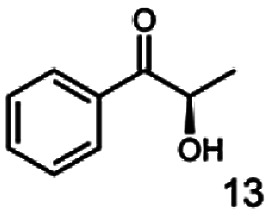	99	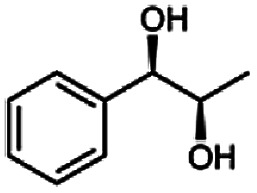	99
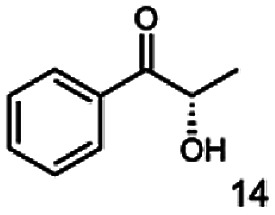	99	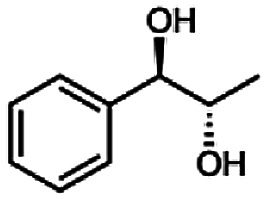	99
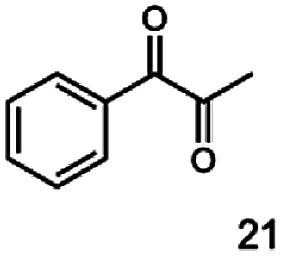	97	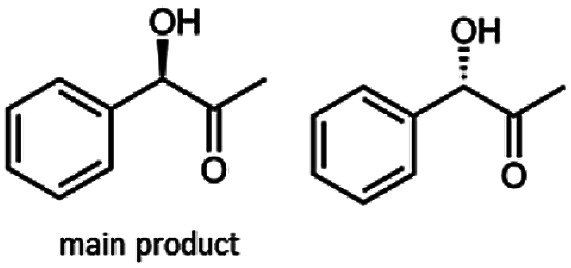	86

At last, the identification of the absolute configuration of the obtained products was investigated. Therefore, the products from the reduction of 3, 9, 7 were produced in preparative-scale biotransformations (200 mM; 48 h; 30 °C; 20 ml), extracted with diethyl ether and after solvent evaporation applied to circular dichroism spectroscopy. Preparative biotransformations of substrate 8 gave the mixed production of 3-hydroxy-2-pentanone as the main product and a small amount of the corresponding diol and was for that reason not further analyzed by CD spectroscopy.

The obtained spectra (Fig. 5) were compared with the CD spectrum of acetoin, which has the closest similar structure. As a result, the produced α-hydroxy ketones could be identified as the (*R*)-enantiomers, because our produced products show the same maximum negative peak at 280 nm as (*R*)-acetoin in CD measurements.^32^ Thus, BcBDH shows the same stereoselectivity for the chosen substrates as for its natural substrate. Surprisingly, the compound 3,4-hexanediol generated with this enzyme gave no CD spectrum. Therefore, we compared the product 3,4-hexanediol obtained by BcBDH with reduction products of the *meso*-BDH from *Bacillus licheniformis* (unpublished data). The reduction of racemic 4-hydroxy-3-hexanone with the *meso*-BDH from *B. licheniformis* led to the synthesis of (*S*,*S*)-3,4-hexanediol and *meso*-3,4-hexanediol. GC analysis revealed different retention times of the diols produced by both BDHs. Because of that we conclude that BcBDH catalyzes the synthesis the (*R*,*R*)-3,4-hexanediol.

**Fig. 4:**
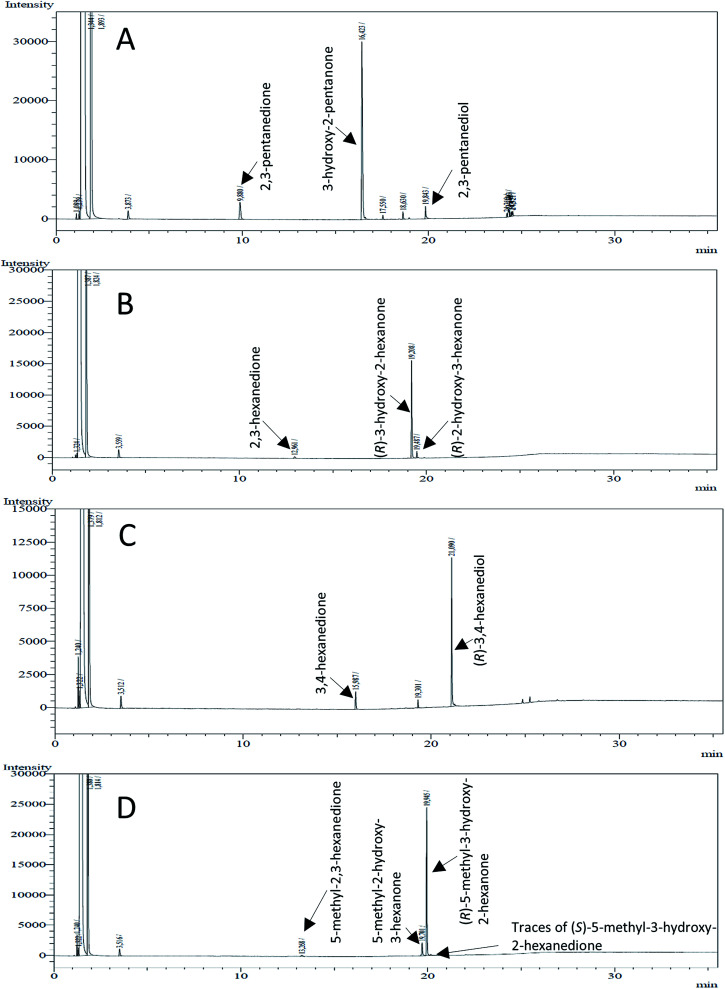
Gas chromatograms of the formation of α-hydroxy ketones and vicinal diols from α-diketones by (*R*,*R*)-BDH of *B. clausii*. Substrates (10 mM each) were reduced by 1 U BDH (activity measured for each specified substrate) using formate (30 mM) and formate dehydrogenase (5 U) to regenerate NADH (0.3 mM) at 30 °C in MES–NaOH buffer (50 mM, pH 6.8; 1 ml). Reaction time 60 min. Biocatalytic reduction of (A) 2,3-pentanedione; (B) 2,3-hexanedione; (C) 3,4-hexanedione; (D) 5-methyl-2,3-hexanedione.

**Fig. 5:**
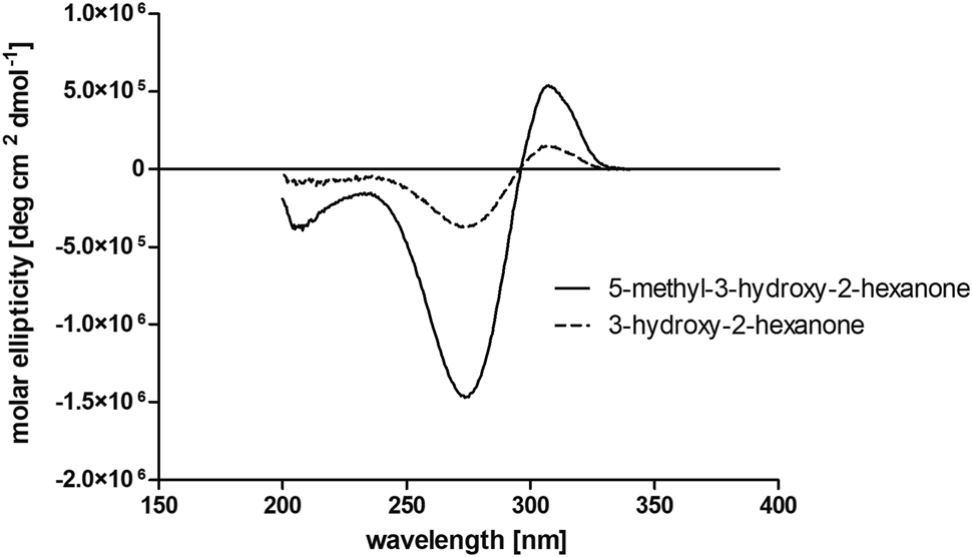
Near UV circular dichroism measurements of the products 5-methyl-3-hydroxy-2-hexanone and 3-hydroxy-2-hexanone obtained by BcBDH catalyzed reduction of 5-methyl-2,3-hexanedione (7) and 2,3-hexanedione (3).

This enzyme showed the same reduction pattern like the previous reported butanediol dehydrogenase BudC from *Serratia marcescens* CECT 977.^19^ The only difference is the conformation of the introduced stereo center. Given that BudC belongs to the SDR superfamily it generates an *S*-configured stereocenter. Whereas BcBDH is a member of the MDR superfamily and introduces an *R*-configured stereocenter. In contrast to that, the reduction of 2,3-pentanediol, 2,3-hexanediol and 3,4-hexanediol with the butanediol dehydrogenase from *Saccharomyces cerevisiae* led exclusively to the corresponding *R*-configured diols.^18^

In conclusion, BcBDH is able to distinguish between asymmetric and symmetric substrate molecules. This leads to a stop of the reduction reaction either at the α-hydroxy ketone intermediate, (in case of asymmetric substrates, like 2,3-hexanedione or 5-methyl-2,3-hexanedione) or the diol (in case of symmetric substrates, like diacetyl or 3,4-hexanedione), respectively. Important to mention is that the keto group next to the phenyl moiety is essential for the reaction to take place. Molecules with such an keto group configuration like, 13, 16, are reduced. In contrast to that PAC and derivatives such as 19, 20 are not accepted by BcBDH (see Section 3.2). We also demonstrated that this enzyme exhibits the same (*R*)-stereoselectivity for unphysiological substrates, like for its natural substrates.

### 3.6 Preparative production of (*R*)-3-hydroxy-2-hexanone using an enzyme membrane reactor

Some α-hydroxy ketones can act as flavouring compounds or as pheromones for insects.^1,4,47,49^ Furthermore, the biocatalytic reduction of 2,3-octanedione by resting cells of *S. cerevisiae* and *B. sulfurescens* could already be demonstrated, yielding the α-hydroxy ketone intermediates that can act as pheromones.^50^ Here, we choose 5-methyl-2,3-hexanedione (7) as a substrate. The corresponding α-hydroxyketone intermediates may act as flavouring compounds.^51^

**Fig. 6:**
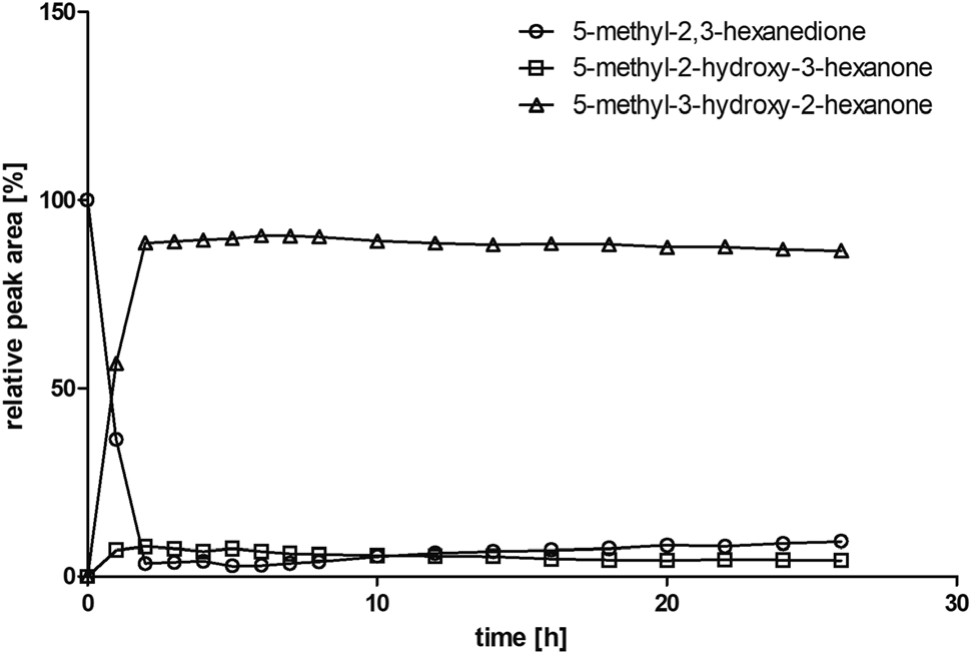
Relative peak area over time for the conversion of 100 mM 5-methyl-2,3-hexanedione with 20 U BcBDH, 0.3 mM NADH, 60 U FDH, 300 mM formate in an enzyme membrane reactor system (10 ml, flow rate 0.2 ml min^−1^) over 26 hours.

Since BcBDH catalyzes the stereoselective synthesis of these compounds, we investigated a continuous stirred tank reactor setup for the conversion of larger volumes. An enzyme membrane reactor (EMR) was applied for this first preparative BcBDH catalyzed biotransformation setup (volume: 300 ml; 5-methyl-2,3-hexanedione: 100 mM). To enhance the solubility of the substrate 5% (v/v) methanol was used, although this was not sufficient to dissolve the substrate completely. But higher methanol concentrations were deleterious for the enzyme. The EMR was operated with 20 U BcBDH, a flow rate of 0.2 ml min^−1^ at 30 °C with gentle stirring (50 rpm) over 26 hours. The reactor outflow was fractionated (10 ml sample) and analyzed by GC (cofactor regeneration see Section 2.15). After an equilibration phase of 2 hours, a conversion of 96% could be achieved, yielding (*R*)-5-methyl-3-hydroxy-2-hexanone as the main product (88% relative peak area) and small amounts of 5-methyl-2-hydroxy-3-hexanone (7% relative peak area). After 26 hours, the conversion declined to 90% (Fig. 6). All in all, a volume of 260 ml substrate solution (30 mmol substrate) was converted which equates to 4 g product.

This experiment is a proof of principle for the application of BcBDH as a biocatalyst in a reactor setup. Further optimization of this system is envisaged.

## 4 Conclusions

We investigated the butanediol dehydrogenase BcBDH from *B. clausii* DSM 8716^T^ for its biocatalytic applicability. BcBDH shows a high stereoselectivity for several α-diketones in addition to its physiological substrates acetoin and diacetyl. Depending on the symmetry of a potential substrate and the length of the aliphatic chain, it is possible to generate selectively the α-hydroxy ketone intermediate or one diol with high stereoselectivity (Table 2). Further, BcBDH accepts molecules with a phenyl moiety as a substrate and reduces these molecules stereoselectively. Surprisingly, this enzyme shows nearly double the activity (U mg^−1^ standard assay) for (*R*)-2-hydroxy-1-phenylpropan-1-on ((*R*)-HPP) compared to acetoin. Moreover, it also reveals good activity for the other here tested “bulky” substrates, which is appealing for the application of this enzyme as a biocatalyst. The good resistance of BcBDH against organic solvents, especially DMSO, underlines the usage of this enzyme for such purposes (Fig. 4). Investigation of the stereoselectivity of BcBDH for several diketones and α-hydroxy ketones revealed the formation of an *R*-configured stereo-center for the main product. Likewise, for its physiological substrates acetoin and diacetyl, this enzyme shows the same stereo-selectivity for several unnatural substrates. BcBDH belongs to the family of zinc-dependent alcohol dehydrogenases. Because of this, we tested the influence of metal ions on the enzyme activity. Even though, this enzyme is zinc-dependent we could observe a high activity boost (more than double the activity) by supplementing this enzyme with Mn^2+^ ions. This effect could also be observed in biotransformation experiments by enhancing the conversion rate by using the enzyme previously pre-incubated with Mn^2+^ ions.

In summary, BcBDH is a suitable biocatalyst for the stereoselective synthesis of α-hydroxy ketones and vicinal diols. With its ability to distinguish between asymmetric and symmetric molecules, the product outcome can be predicted. Furthermore, the ability of accepting “bulky” substrates and the stereoselective reduction of these molecules makes this enzyme even more appealing.

## Conflicts of interest

There are no conflicts to declare.

## Supplementary Material

RA-010-D0RA02066D-s001
