# Supplementary material for: Synthesis of α-hydroxy ketones and vicinal (R,R)-diols by Bacillus clausii DSM 8716T butanediol dehydrogenase
Source: RSC Adv. 2020 Mar 25;10(21):12206–16. doi: 10.1039/d0ra02066d (PMC9050739; doi:10.1039/d0ra02066d)
Supplement: RA-010-D0RA02066D-s001 [file RA-010-D0RA02066D-s001.pdf]

Electronic Supplementary Material (ESI) for RSC Advances.
This journal is © The Royal Society of Chemistry 2020

Electronic Supplementary Information

Synthesis of α-hydroxy ketones and vicinal (*R*,*R*)-diols by *Bacillus clausii* DSM 8716^T butanediol dehydrogenase

Lukas Muschallik[a], Denise Molinnus[a], Melanie Jablonski[a], Carina Ronja Kipp[a], Johannes Bongaerts[a], Martina Pohl[b], Torsten Wagner[a], Michael J. Schöning[a], Thorsten Selmer[a], Petra Siegert*[a]

[a] *Institute of Nano- and Biotechnologies, Aachen University of Applied Sciences, 52428 Jülich, Germany*

[b] *IBG-1: Biotechnology, Forschungszentrum Jülich, 52425 Jülich, Germany*

*Corresponding author at: Heinrich-Mussmannstr. 1, 52428 Jülich, Germany. *E-mail address:* siegert@fh-aachen.de

Table of contents

1. GC-MS spectra
2. GC-Chromatograms
3. NMR-spectrum

1 GC-MS spectra

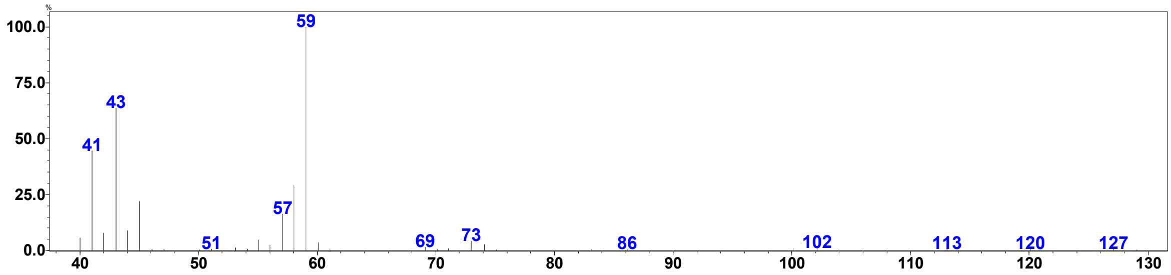

Supplementary Figure 1: Mass spectrum of 3–hydroxy-2-pentanone.Retention time: 16.4 min.

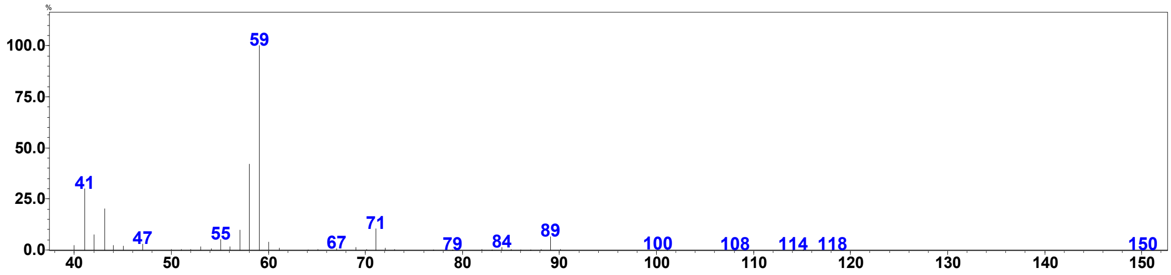

Supplementary Figure 2: Mass spectrum of (*R*)-3,4-hexanediol. Retention time: 21.0 min.

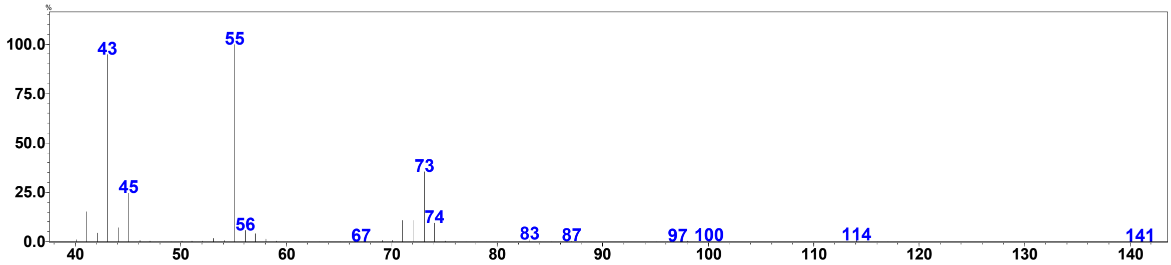

Supplementary Figure 3: Mass spectrum of (*R*)-3-hydroxy-2-hexanone. Retention time: 19.3 min.

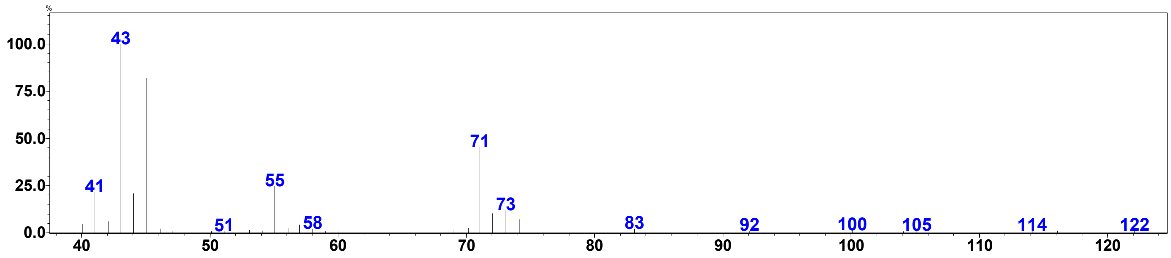

Supplementary Figure 4: Mass spectrum of (*R*)-2-hydroxy-3-hexanone. Retention time: 19.4 min.

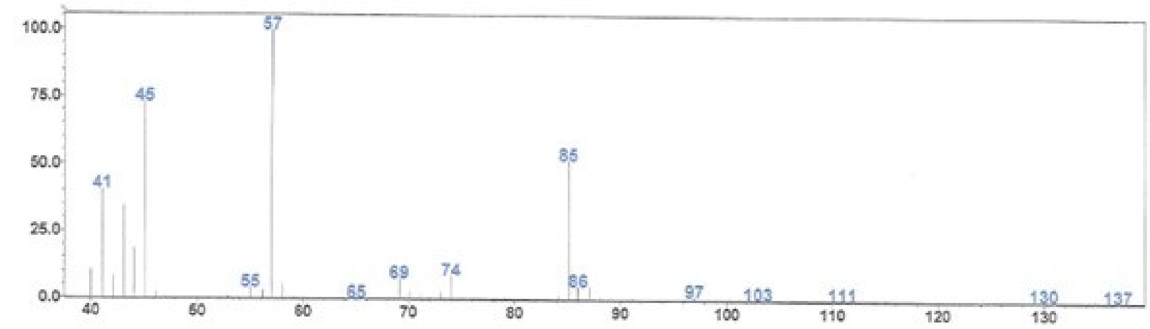

Supplementary Figure 5: Mass spectrum of (*R*)-2-hydroxy-3-hexanone. Retention time: 19.7 min.

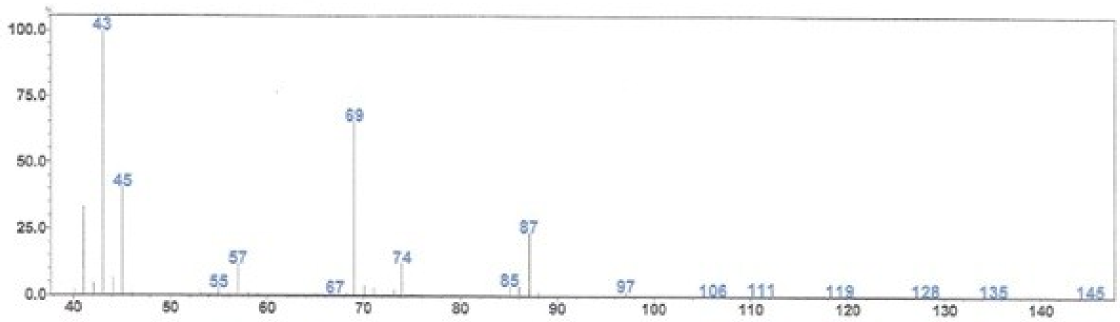

Supplementary Figure 6: Mass spectrum of (*R*)-2-hydroxy-3-hexanone. Retention time: 19.9 min.

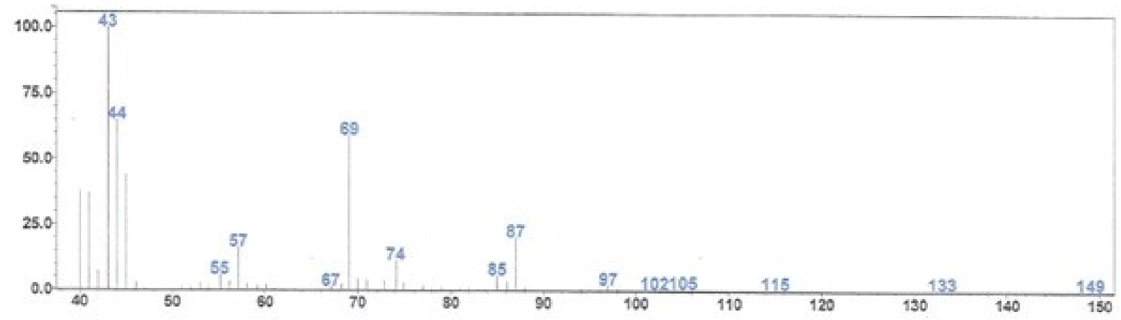

Supplementary Figure 7: Mass spectrum of (*S*)-3-hydroxy-2-hexanone. Retention time: 20.1 min.

2 GC-Chromatogramms

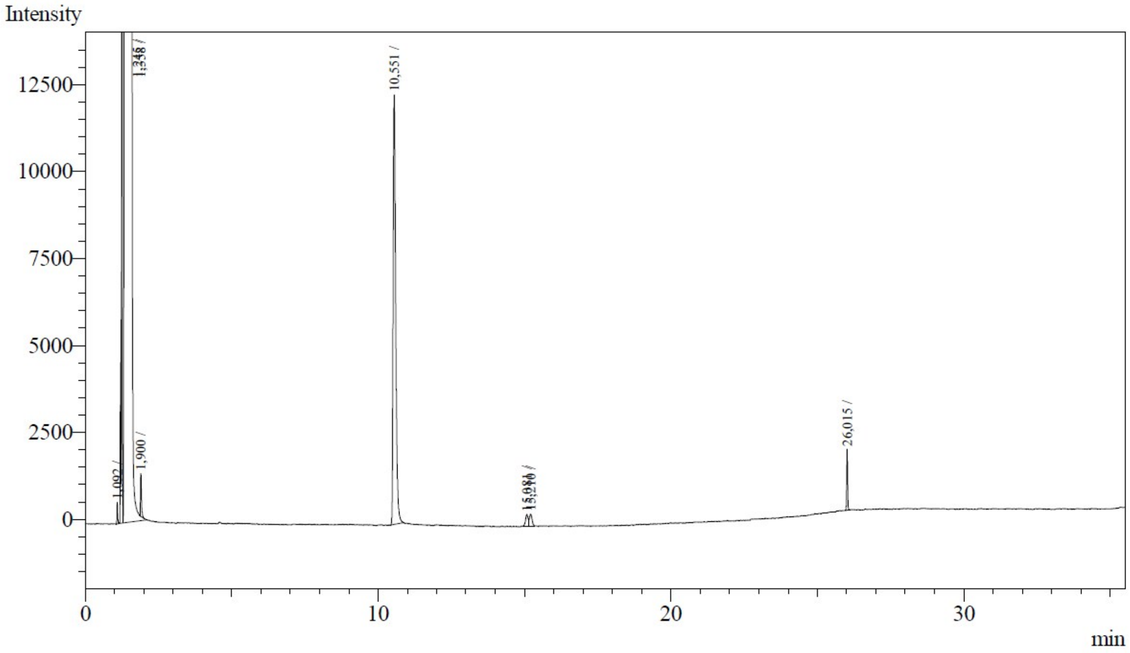

Supplementary Figure 8: Gas chromatogramm of the reduction of 3-hydroxy-3-methyl-2-butanone by BclBDH. Substrate (10 mM each) was reduced by 1 U BDH using formate (30 mM) and formate dehydrogenase (5 U) to regenerate NADH (0.3 mM) at 30 °C in MES-NaOH buffer (50 mM, pH 6.8; 1 ml). Reaction time 0 min.

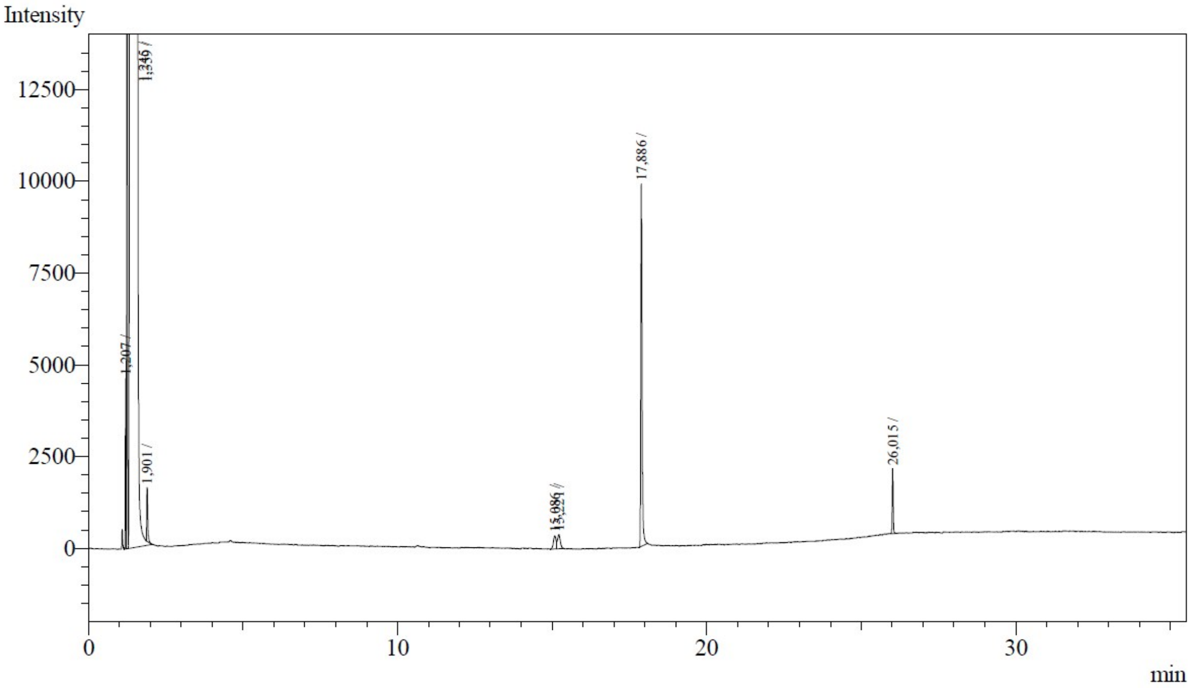

Supplementary Figure 9: Gas chromatogramm of the reduction of 3-hydroxy-3-methyl-2-butanone by BcBDH. Substrate (10 mM each) was reduced by 1 U BDH using formate (30 mM) and formate dehydrogenase (5 U) to regenerate NADH (0.3 mM) at 30 °C in MES-NaOH buffer (50 mM, pH 6.8; 1 ml). Reaction time 60 min.

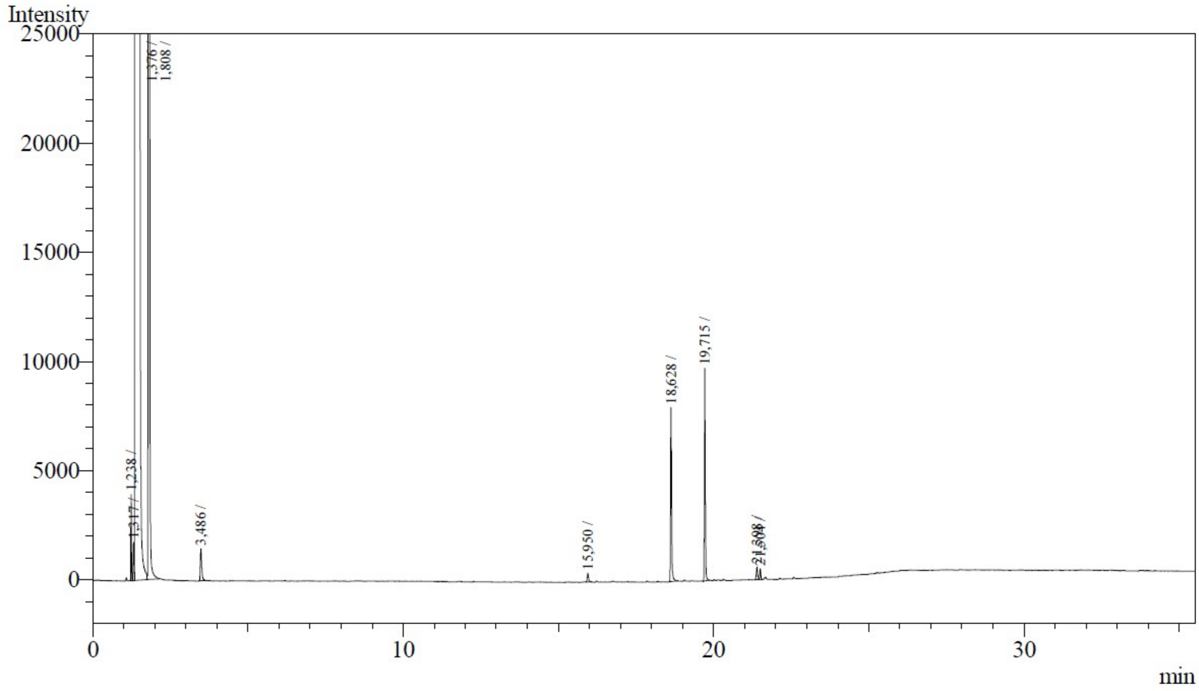

Supplementary Figure 10*:* Gas chromatogramm of the reduction of *Rac* 4-hydroxy-3-hexanone by BcBDH. Substrate (10 mM each) was reduced by 1 U BDH using formate (30 mM) and formate dehydrogenase (5 U) to regenerate NADH (0.3 mM) at 30 °C in MES-NaOH buffer (50 mM, pH 6.8; 1 ml). Reaction time 0 min.

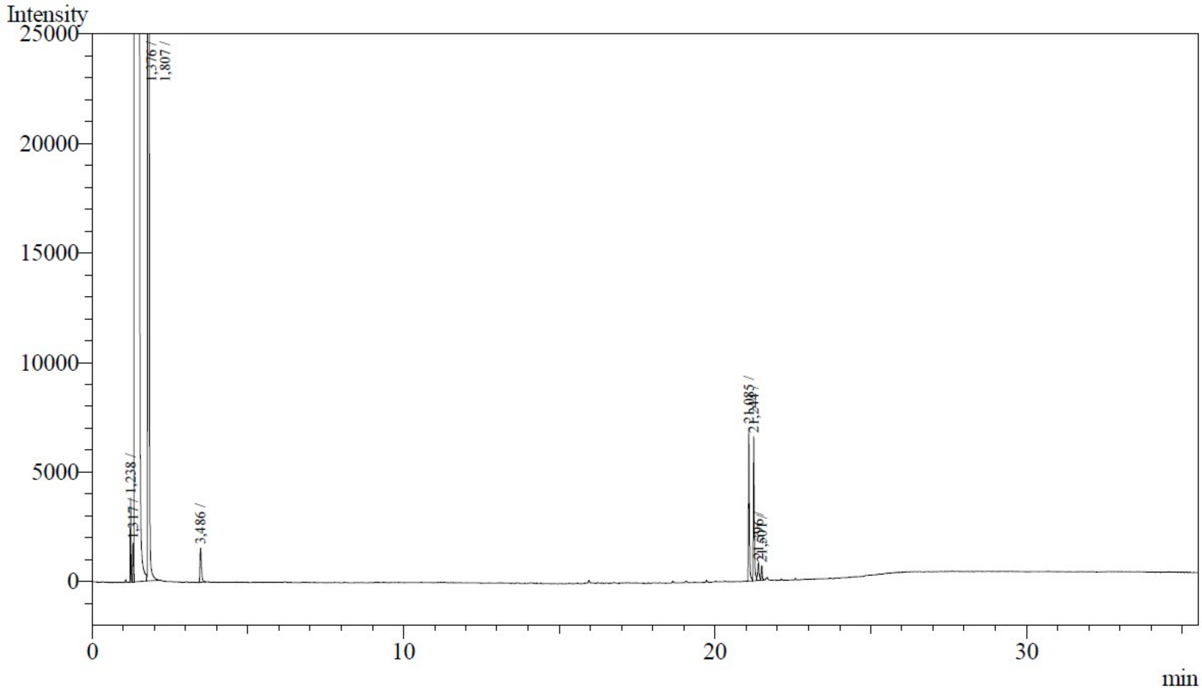

Supplementary Figure 11: Gas chromatogramm of the reduction of *Rac* 4-hydroxy-3-hexanone by BcBDH. Substrate (10 mM each) was reduced by 1 U BDH using formate (30 mM) and formate dehydrogenase (5 U) to regenerate NADH (0.3 mM) at 30 °C in MES-NaOH buffer (50 mM, pH 6.8; 1 ml). Reaction time 60 min.

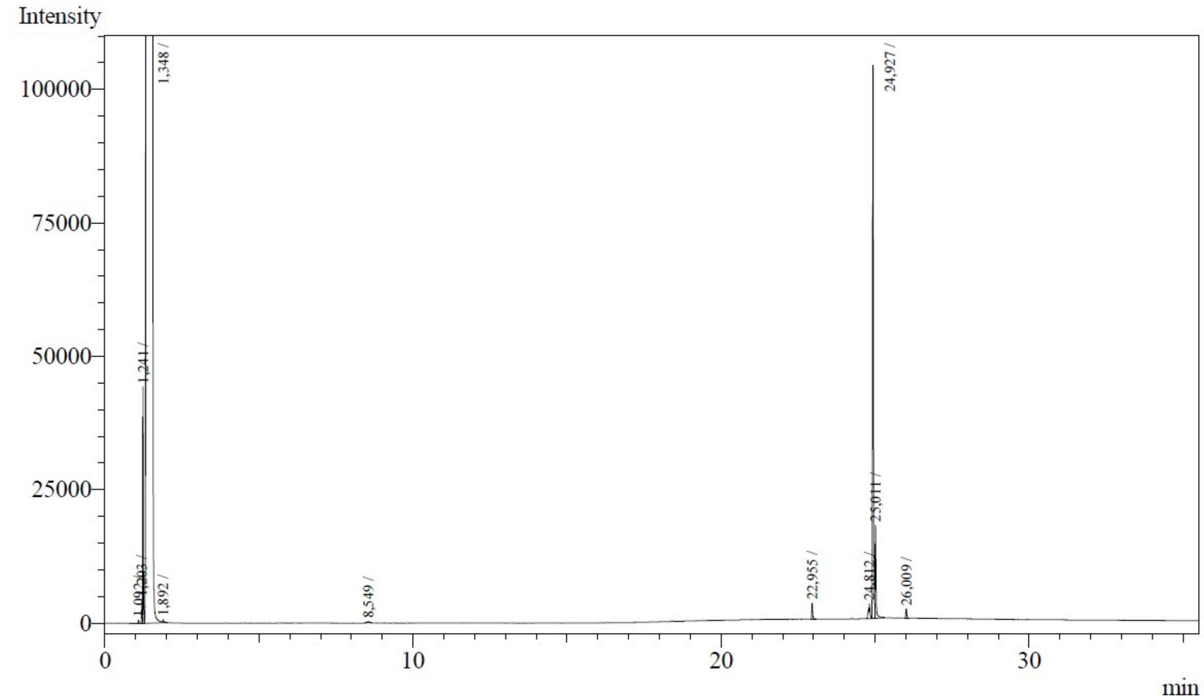

Supplementary Figure 12: Gas chromatogramm of the reduction of (*S*)-HPP by BcBDH. Substrate (10 mM each) was reduced by 1 U BDH using formate (30 mM) and formate dehydrogenase (5 U) to regenerate NADH (0.3 mM) at 30 °C in MES-NaOH buffer (50 mM, pH 6.8; 1 ml). Reaction time 0 min.

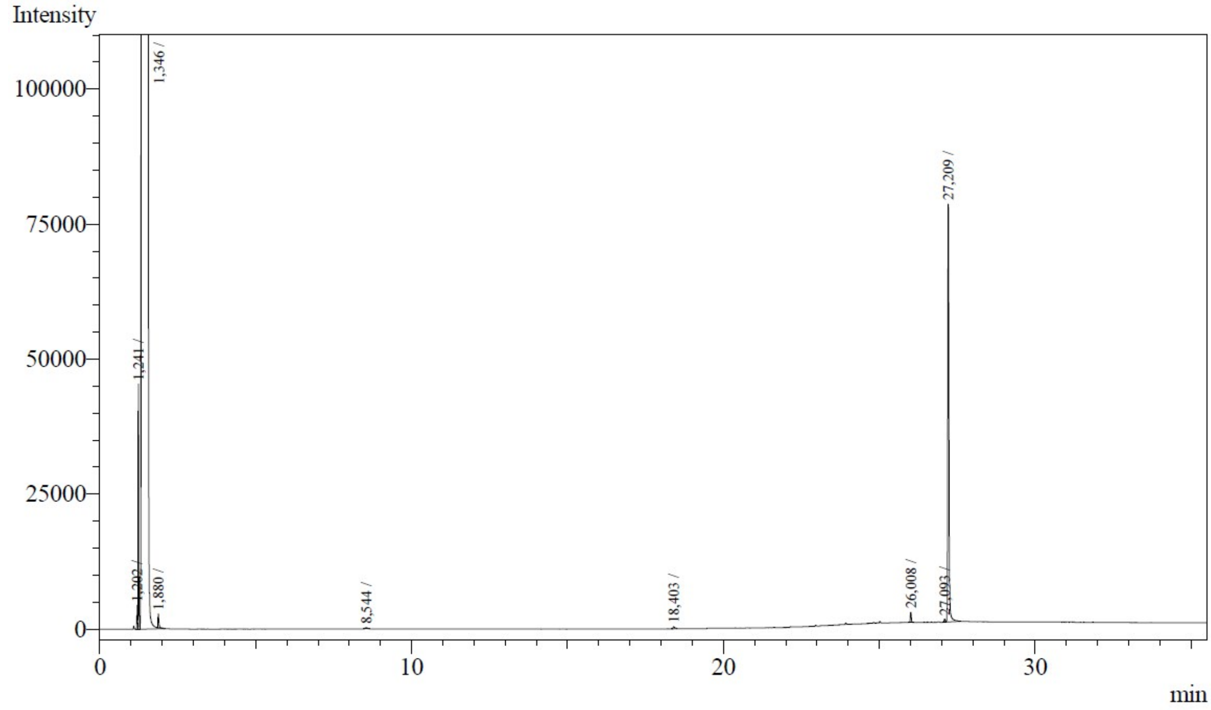

Supplementary Figure 13: Gas chromatogramm of the reduction of (*S*)-HPP by BcBDH. Substrate (10 mM each) was reduced by 1 U BDH using formate (30 mM) and formate dehydrogenase (5 U) to regenerate NADH (0.3 mM) at 30 °C in MES-NaOH buffer (50 mM, pH 6.8; 1 ml). Reaction time 60 min.

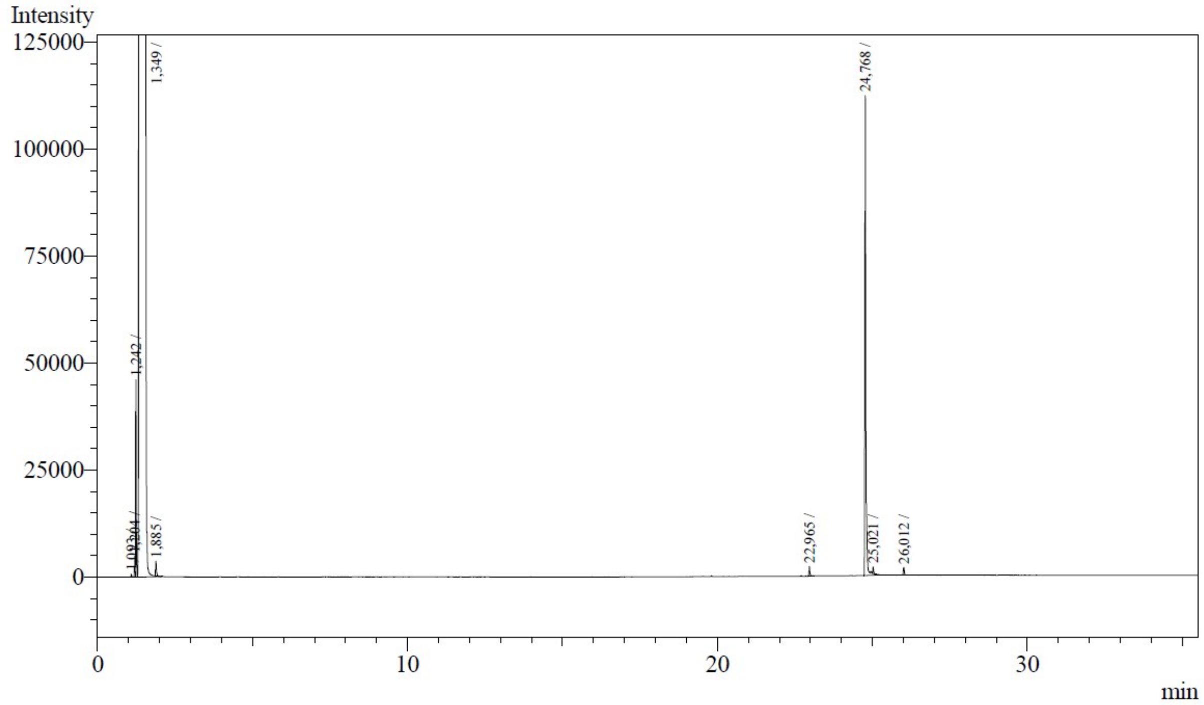

Supplementary Figure 14: Gas chromatogramm of the reduction of (*R*)-HPP by BcBDH. Substrate (10 mM each) was reduced by 1 U BDH using formate (30 mM) and formate dehydrogenase (5 U) to regenerate NADH (0.3 mM) at 30 °C in MES-NaOH buffer (50 mM, pH 6.8; 1 ml). Reaction time 0 min.

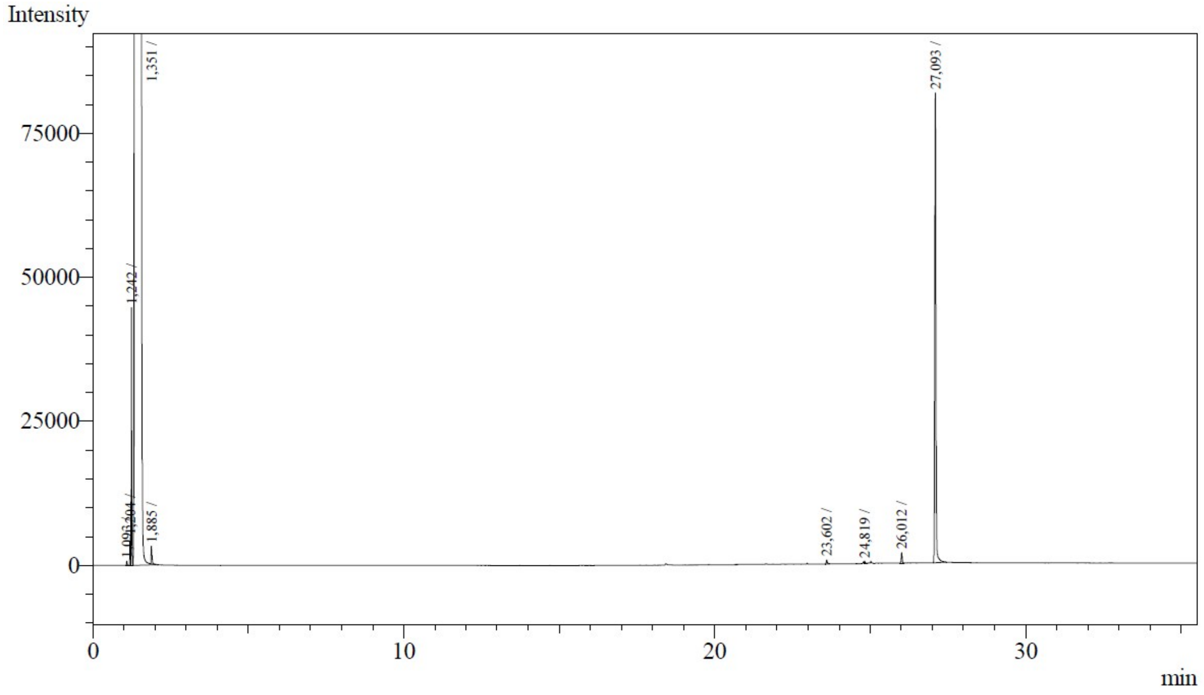

Supplementary Figure 15: Gas chromatogramm of the reduction of (*R*)-HPP by BcBDH. Substrate (10 mM each) was reduced by 1 U BDH using formate (30 mM) and formate dehydrogenase (5 U) to regenerate NADH (0.3 mM) at 30 °C in MES-NaOH buffer (50 mM, pH 6.8; 1 ml). Reaction time 60 min.

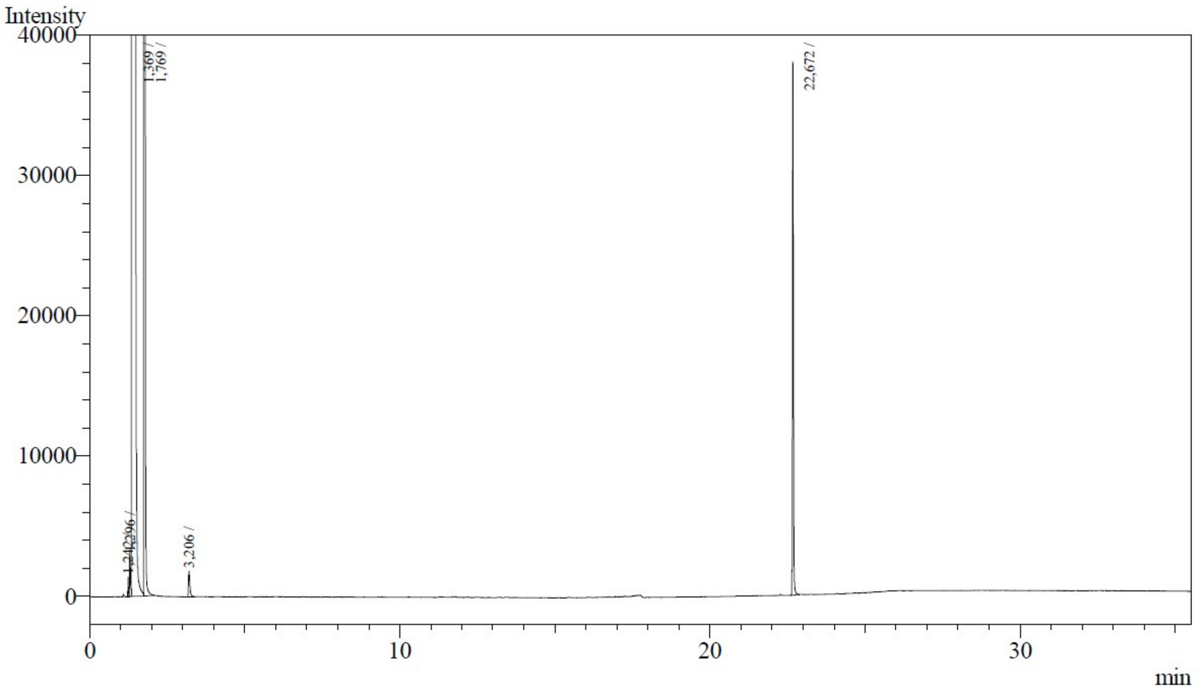

Supplementary Figure 16: Gas chromatogramm of the reduction of 1-phenyl-1,2-propanedione by BcBDH. Substrate (10 mM each) was reduced by 1 U BDH using formate (30 mM) and formate dehydrogenase (5 U) to regenerate NADH (0.3 mM) at 30 °C in MES-NaOH buffer (50 mM, pH 6.8; 1 ml). Reaction time 0 min.

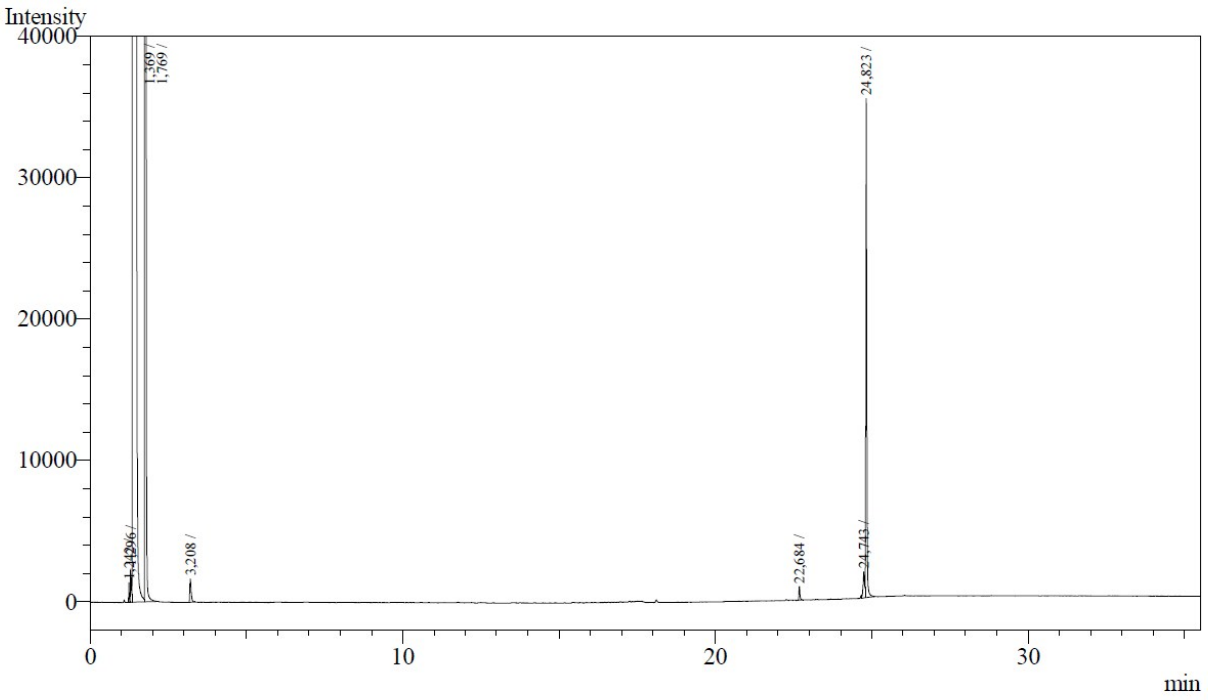

Supplementary Figure 17: Gas chromtogramm of the reduction of 1-phenyl-1,2-propanedione by BcBDH. Substrate (10 mM each) was reduced by 1 U BDH using formate (30 mM) and formate dehydrogenase (5 U) to regenerate NADH (0.3 mM) at 30 °C in MES-NaOH buffer (50 mM, pH 6.8; 1 ml). Reaction time 60 min.

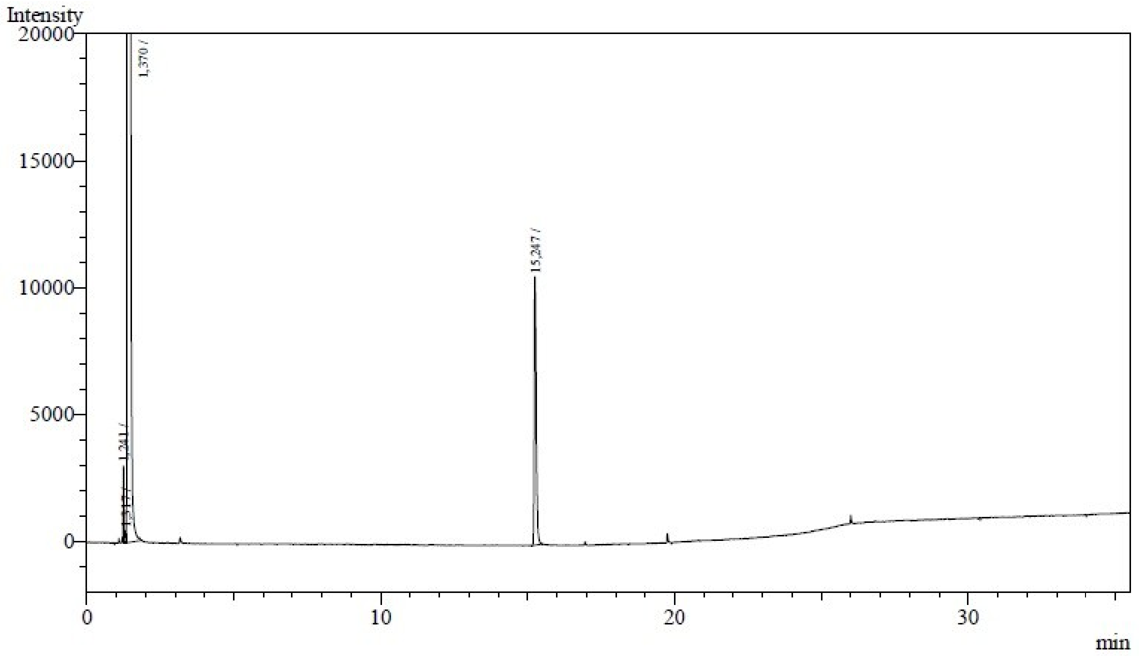

Supplementary Figure 18: Gas chromatogramm of the reduction of 2,3-heptanedione by BcBDH. Substrate (10 mM each) was reduced by 1 U BDH using formate (30 mM) and formate dehydrogenase (5 U) to regenerate NADH (0.3 mM) at 30 °C in MES-NaOH buffer (50 mM, pH 6.8; 1 ml). Reaction time 0 min.

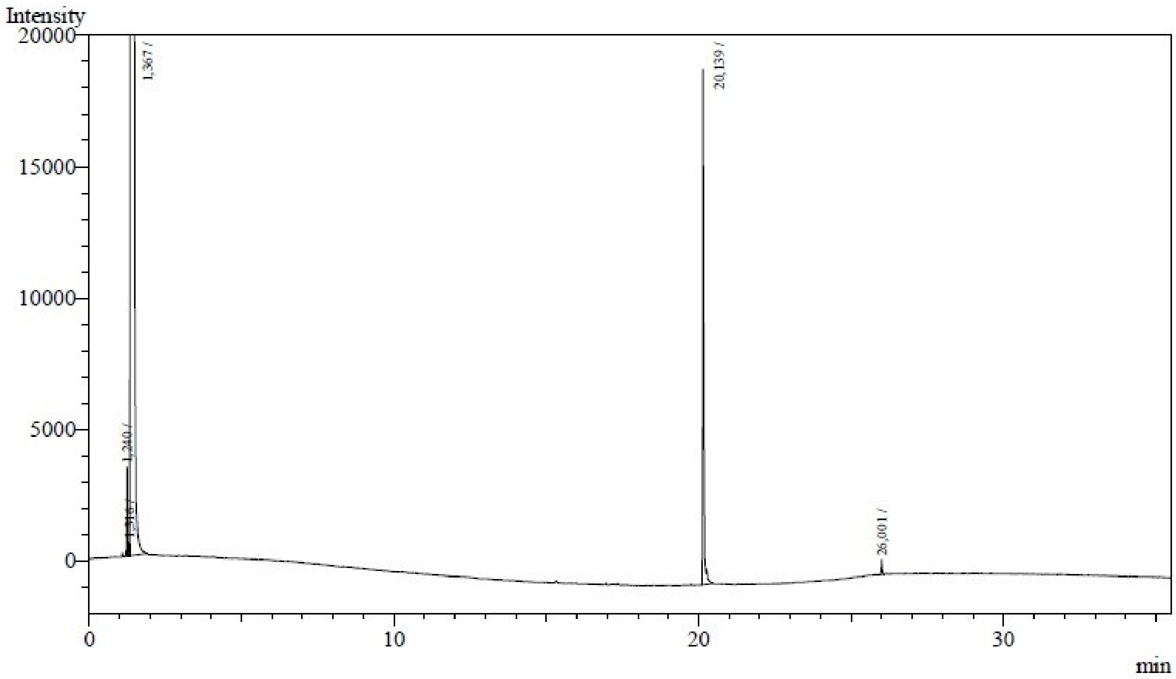

Supplementary Figure 19: Gas chromatogramm of the reduction of 2,3-heptanedione by BcBDH. Substrate (10 mM each) was reduced by 1 U BDH using formate (30 mM) and formate dehydrogenase (5 U) to regenerate NADH (0.3 mM) at 30 °C in MES-NaOH buffer (50 mM, pH 6.8; 1 ml). Reaction time 60 min.

3 NMR

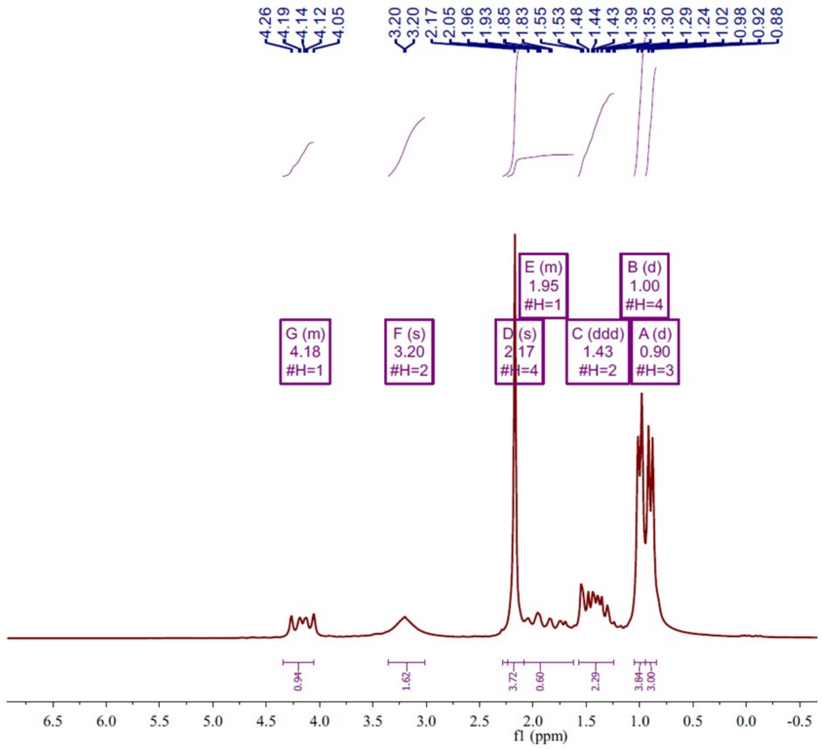

Supplementary Figure 20: ^{1}H-NMR of biocatalytic product (*R*)-5-methyl-3-hydroxy-2-hexanone, containing small amounts of 5-methyl-2-hydroxy-3-hexanone. ($CDCl_3$) σ 0.90 (d, 3H), 1,00 (d, 4H), 1.43 (ddd, 2H), 1.95 (m, 1H), 2.17 (s, 4H), 3.20 (s, 2H 4.18 (m, 1H). s = singlet; d = doublet; ddd = doublet of doublet of doublet; m = multiplet.

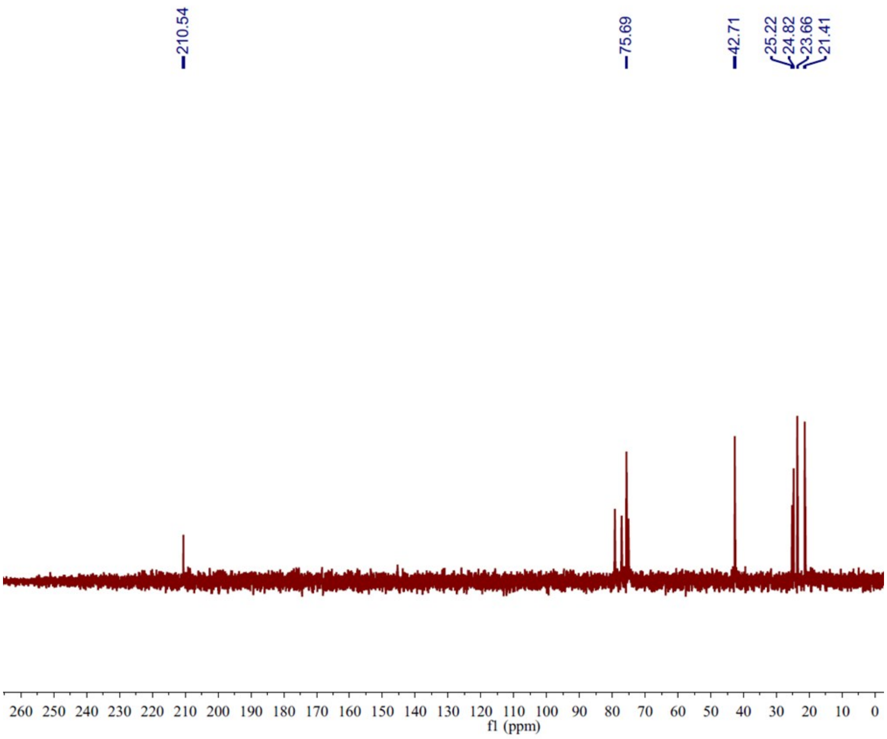

Supplementary Figure 21: ^{13}C-NMR of biocatalytic product (*R*)-5-methyl-3-hydroxy-2-hexanone, containing small amounts of 5-methyl-2-hydroxy-3-hexanone. (CDCl3) σ 21.41; 23.66; 24.82; 25.22; 42.71; 75.69; 210.54.
